# Polycystin-2 is an essential ion channel subunit in the primary cilium of the renal collecting duct epithelium

**DOI:** 10.7554/eLife.33183

**Published:** 2018-02-14

**Authors:** Xiaowen Liu, Thuy Vien, Jingjing Duan, Shu-Hsien Sheu, Paul G DeCaen, David E Clapham

**Affiliations:** 1 Department of CardiologyHoward Hughes Medical Institute, Boston Children's HospitalBostonUnited States; 2 Department of NeurobiologyHarvard Medical SchoolBostonUnited States; 3 Department of PharmacologyNorthwestern University, Feinberg School of MedicineChicagoUnited States; 4 Department of PathologyBoston Children’s HospitalBostonUnited States; Stanford University School of MedicineUnited States

**Keywords:** polycystin-2, primary cilia, polycystin-1, plasma membrane, Mouse

## Abstract

Mutations in the polycystin genes, *PKD1* or *PKD2,* results in Autosomal Dominant Polycystic Kidney Disease (ADPKD). Although a genetic basis of ADPKD is established, we lack a clear understanding of polycystin proteins’ functions as ion channels. This question remains unsolved largely because polycystins localize to the primary cilium – a tiny, antenna-like organelle. Using a new ADPKD mouse model, we observe primary cilia that are abnormally long in cells associated with cysts after conditional ablation of *Pkd1* or *Pkd2*. Using primary cultures of collecting duct cells, we show that polycystin-2, but not polycystin-1, is a required subunit for the ion channel in the primary cilium. The polycystin-2 channel preferentially conducts K^+^ and Na^+^; intraciliary Ca^2+^, enhances its open probability. We introduce a novel method for measuring heterologous polycystin-2 channels in cilia, which will have utility in characterizing *PKD2* variants that cause ADPKD.

## Introduction

Autosomal dominant polycystic kidney disease (ADPKD) is an adult-onset disease characterized by focal cyst development resulting from heterozygous mutations in *PKD1* or *PKD2* (Brasier and Henske, 1997; Grantham, 2001; Hughes et al., 1995; Mochizuki et al., 1996). While considered a dominant monogenic disease, the prevailing two-hit model states that ADPKD is recessive at the cellular level and that cysts develop from cells after acquiring a second somatic mutation to deactivate the remaining normal allele (Koptides et al., 1999; Pei, 2001; Qian et al., 1997; Wu et al., 1998). Mouse models of ADPKD implicate ciliary polycystin-1 and polycystin-2 dysfunction in kidney cyst formation. Complete genetic knockout of either *Pkd1* or *Pkd2* in mice results in embryonic lethality due to structural defects in the cardiovascular system, pancreas, and kidneys (Boulter et al., 2001; Kim et al., 2000; Lu et al., 1997; Wu and Somlo, 2000; Wu et al., 2002). The onset of kidney cyst development in adult mice following conditional inactivation of *Pkd1* or the intraflagellar transport protein kinesin, KIF3a (required for cilia formation), progresses well into adulthood, in analogy to the late progression of ADPKD in humans (Davenport et al., 2007; Piontek et al., 2007; Shibazaki et al., 2008). Conditional repression of either *Pkd1* (*Pax8^rtTA^; TetO-cre; Pkd1^fl/fl^*) (Shibazaki et al., 2008) or *Pkd2* (*Pax8^rtTA^; TetO-cre; Pkd2^fl/fl^*) (Ma et al., 2013) results in kidney cysts within 10 weeks after the start of doxycycline induction, suggesting that expression of both genes is necessary to prohibit cyst development in mature mice. Recently, the cystic phenotype found in mice deficient in *Pkd2* can be dose-dependently rescued by *Pkd2* transgene expression (Li et al., 2015). Our poor understanding of the functional properties of polycystin-1, polycystin-2, and the polycystin-1/polycystin-2 complex impedes the development of therapeutic strategies; ADPKD is currently treated by dialysis and kidney transplant (LaRiviere et al., 2015). Since polycystin-2’s function is unclear, it is not currently known if all ADPKD-causing variants in *Pkd2* cause a loss of function of the putative ion channel in primary cilia.

Polycystin-1 (PC1) is predicted to adopt an 11-transmembrane topology with a large autocleaved (G protein-coupled receptor proteolytic site, GPS) amino-terminal ectodomain (>3000 residues)(Harris et al., 1995) that is comprised of an array of putative adhesion and ligand-binding modules (Burn et al., 1995; Hughes et al., 1995; Qian et al., 2002). Polycystin-2 (or PC2, TRPP1, formerly TRPP2; encoded by *PKD2*) is a member of the large, 6-transmembrane spanning transient receptor potential (TRP) ion channel family (Ramsey et al., 2006; Venkatachalam and Montell, 2007) and has been observed to form a complex with polycystin-1 (*PKD1* gene). It is proposed to interact with polycystin-2 through a probable coiled-coil domain (Newby et al., 2002; Qian et al., 1997; Tsiokas et al., 1997), but in other experiments, the polycystin-1 and polycystin-2 interaction is preserved in overexpressed systems without the coiled-coil domain and is dependent on the N-terminal domain (Babich et al., 2004; Ćelić et al., 2012; Feng et al., 2008). However, the cryo-EM structure of purified polycystin-2 in lipid nanodiscs forms a homotetramer, with and without its C-terminal coiled-coil and N-terminal domains (Shen et al., 2016). Based on biochemistry and immunoreactivity, both proteins can be found in the primary cilium and ER (Ong and Wheatley, 2003; Yoder et al., 2002). In addition, some studies suggest that polycystin-1 and polycystin-2 may reciprocally affect each other’s surface membrane or ciliary localization (Harris et al., 1995; Ong and Wheatley, 2003; Xu et al., 2007a). A recent study using inner medullary collecting duct (IMCD) cell lines derived from human ADPKD cysts suggests that impairing the function of polycystin-1 or polycystin-2 negatively affects the localization of the other polycystin: cells expressing an ADPKD-associated polycystin-1 mutation that prevents GPS domain cleavage have decreased amounts of both polycystin-1 and polycystin-2 in their primary cilia (Xu et al., 2007a). Our understanding of ADPKD pathogenesis is hampered by disagreements about the basic properties of the putative polycystin-2 current. Furthermore, little is understood regarding what role, if any, the primary cilia have in controlling the progression of cyst formation in ADPKD. Nonetheless, there is no ambiguity in the finding that mutations in *PKD1* or *PKD2* are genetically linked to formation of cysts in kidney and other tissues to cause significant morbidity and mortality in humans (Mochizuki et al., 1996; Ong and Harris, 2015).

Previous work reported single channel events from exogenously expressed polycystin-1 and polycystin-2 from the plasma membrane and in reconstituted polycystin-1 and polycystin-2 proteins recorded in formulated bilayers were attributed to an polycystin-1/polycystin-2 ion channel complex (Delmas et al., 2004; González-Perrett et al., 2001; Hanaoka et al., 2000). However, these non-ciliary preparations produced contradictory findings regarding its ion selectivity and voltage dependence: the polycystin-2 ion channel was initially reported to conduct calcium (González-Perrett et al., 2001; Hanaoka et al., 2000), and then not conduct calcium (Cai et al., 2004). Most recently, a gain-of-function mutation (F604P), but not *wt* polycystin-2, underlies a measurable current when heterologously expressed in *Xenopus* oocytes (Arif Pavel et al., 2016). This study demonstrated that plasma membrane polycystin-2 expression does not appear to be hampered by the lack of polycystin-1, but rather that native polycystin-2 channels appears to be constitutively closed unless mutated (F604P; affecting flexibility of the S5 segment as in TRPML1). The monovalent-selective current of this mutant is blocked by divalent ions (Ca^2+^ and Mg^2+^). As we will review in the Discussion, several putative polycystin-2 activators have been reported to sensitize polycystin-2 channels, but we have been unable to reproduce these results (Kim et al., 2016; Leuenroth et al., 2007). While the apparent differences observed can be rooted in methodology, these preparations are measured from non-ciliary membranes and thus share the same disadvantage. Recently, two methods have been used to measure ion currents from intact primary cilia (DeCaen et al., 2013; Kleene and Kleene, 2012), thus preserving their unique native microenvironment without the need for reconstitution.

For this study, we crossed our *Arl13b-EGFP^tg^* strain (DeCaen et al., 2013) with *Pax8^rtTA^; TetO-cre; Pkd1^fl/fl^* (*cPkd1*) or *Pax8^rtTA^; TetO-cre; Pkd2^fl/fl^* (*cPkd2*) mice provided by the Somlo lab (Ma et al., 2013; Shibazaki et al., 2008). The progeny express the *Arl13b-EGFP^tg^* cilia reporter, and ablation of either *Pkd1* or *Pkd2* genes expression in the kidney under the Pax8^rtTA^ promotor (Traykova-Brauch et al., 2008) is *TetO-cre* doxycycline-dependent. For brevity, we will call these animal strains either *Arl13b-EGFP^tg^:cPkd1* or *Arl13b-EGFP^tg^:cPkd2*, respectively. Consistent with previous reports (Ma et al., 2013), we find that repression of either *Pkd1* or *Pkd2* results in obvious kidney cysts within two months after removal of doxycycline. Primary cilia from cysts of either doxycycline-treated *Arl13b-EGFP^tg^:cPkd1* or *Arl13b-EGFP^tg^:cPkd2* mice were substantially elongated compared to control littermates. We utilized the cilium patch method to directly measure ciliary ion channels from primary cultures of inner medullary collecting duct epithelial cells (pIMCD) from these mice. We characterize the native ciliary polycystin-2 currents, which can be conditionally ablated using doxycycline in the *Arl13b-EGFP^tg^:cPkd2* mouse model.

Surprisingly, polycystin-2 forms a functional ion channel in primary cilia without polycystin-1 expression, calling into question the hypothesis that polycystin-1 is an obligate subunit of putative polycystin-1/polycystin-2 heteromeric channel complex. The ciliary polycystin-2 current preferentially conducts the monovalents K^+^ and Na^+^, over divalent Ca^2+^ ions. Millimolar external [Ca^2+^] weakly permeates through the polycystin-2 pore and blocks the inward sodium current. The open probability of polycystin-2 is enhanced by internal calcium (EC_50_ = 1.3 μM), slightly exceeding the resting cilioplasmic [Ca^2+^] (~300–600 nM) (Delling et al., 2013, 2016). Native constitutive plasma membrane currents are not affected by conditional ablation of either *Pkd1* or *Pkd2* from pIMCD cells. Thus, we find no evidence for homomeric or heteromeric polycystin channels in the plasma membrane. Heterologous, stably-expressed polycystin-2-GFP traffics to the primary cilia of HEK-293 cells, where cilia patch clamp recordings recapitulate the ion selectivity and internal calcium potentiation effects observed in primary cilia of native pIMCD cells.

## Results

### Progressive cyst formation in a new mouse model

**Figure 1.**
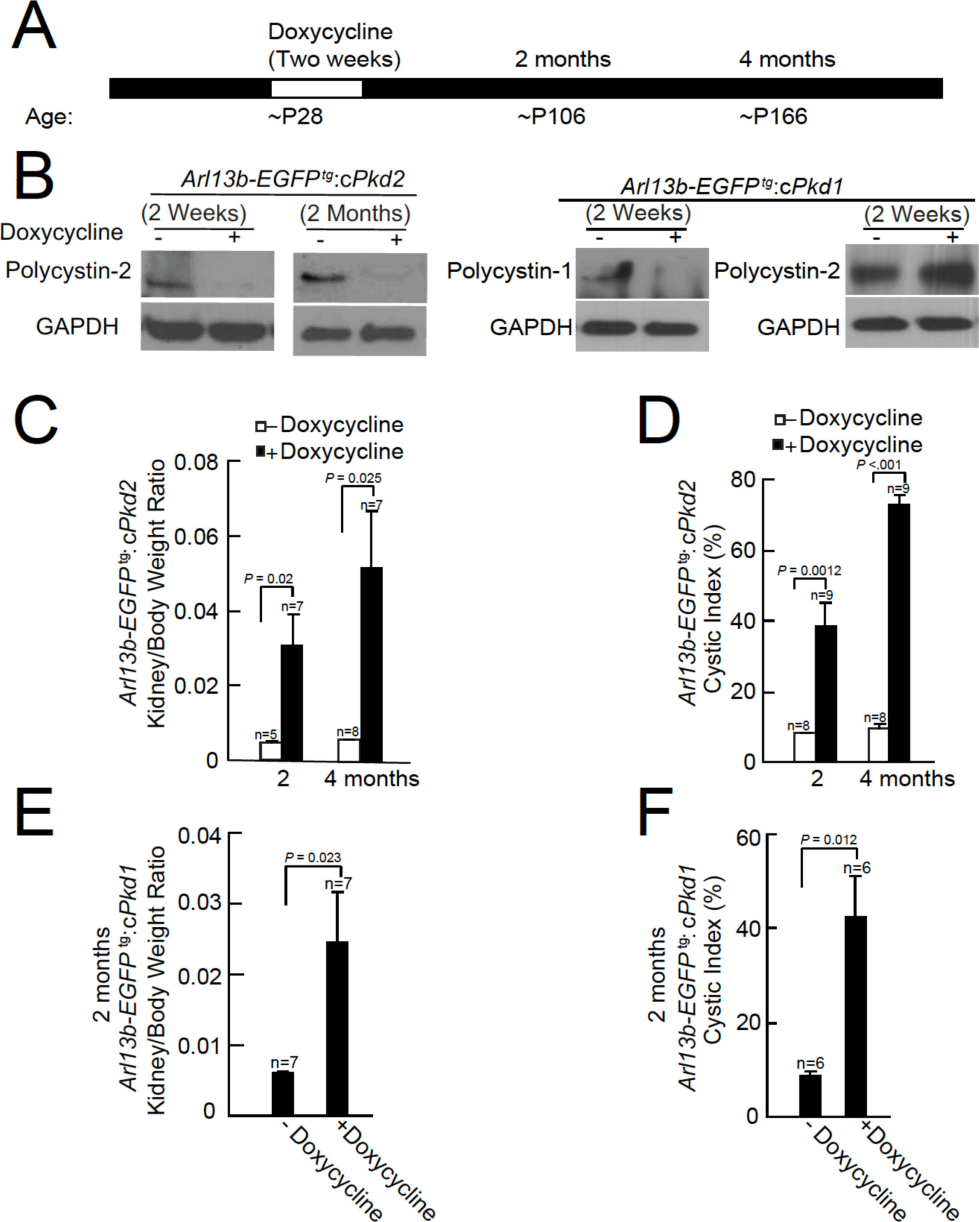
Onset of kidney tubule cyst formation in *Arl13b-EGFP^tg^:cPkd1* and *Arl13b-EGFP^tg^:cPkd2* animals. (**A**) Study design to assess cyst formation after genetic ablation of either *Pkd1* or *Pkd2*. One protocol is shown that assesses polycystin-1 or polycystin-2 at P28 in the conditional knockout mouse. (**B**) Loss of polycystin-1 and polycystin-2 protein expression as assessed by immunoblot, 2 weeks and 2 months after doxycycline removal. Whole cell lysates were prepared from pIMCD cells and subjected to western blot analysis (three independent experiments). A trace amount of polycystin-2 (first row, faint band at 2 weeks after doxycycline removal) is similar to that previously reported (Ma et al., 2013). The band at the left edge (c*Pkd1*, 2 weeks + Doxycycline, first row), appears to be nonspecific, although we cannot rule out polycystin-1 contamination from non-tubule cells. (**C**) Kidney weight/body was increased in *Arl13b-EGFP^tg^:cPkd2* mice with doxycycline treatment compared to control littermates without doxycycline treatment. (**D**) Cystic index (Materials and methods) shows that cysts increased in *Arl13b-EGFP^tg^:cPkd2* mice. (**E**) Kidney weight/body was increased in *Arl13b-EGFP^tg^:cPkd1* with doxycycline treatment (2 months after doxycycline removal) compared to control littermates without doxycycline treatment. (**F**) Cystic index shows increased size and number of cysts in *Arl13b-EGFP^tg^:cPkd1* mice.

Previous work demonstrated that the human ADPKD kidney cyst phenotype can be reproduced in mice 14 weeks after conditional ablation of nephron-localized *Pkd1* or *Pkd2 *(Ma et al., 2013). To understand the putative ciliary ion channel function of polycystin-1 and/or polycystin-2, and to determine the effects of kidney cyst formation on cilia morphology, we crossed our *Arl13b-EGFP^tg^* strain (DeCaen et al., 2013) with *cPkd1* or *cPkd2* mice (provided by S. Somlo Yale Univ.). We then induced either *Pkd1* or *Pkd2* gene inactivation in adult animals (~P28) by introducing doxycycline (2 mg/ml or 3.9 mM) into the drinking water for two weeks. After this treatment period, doxycycline was removed and kidney histology was performed from 2 and 4 month post-treatment animals (Figure 1A, Figure 1—figure supplement 1, Figure 1—figure supplement 2A,B).

**Figure 1—figure supplement 1.**
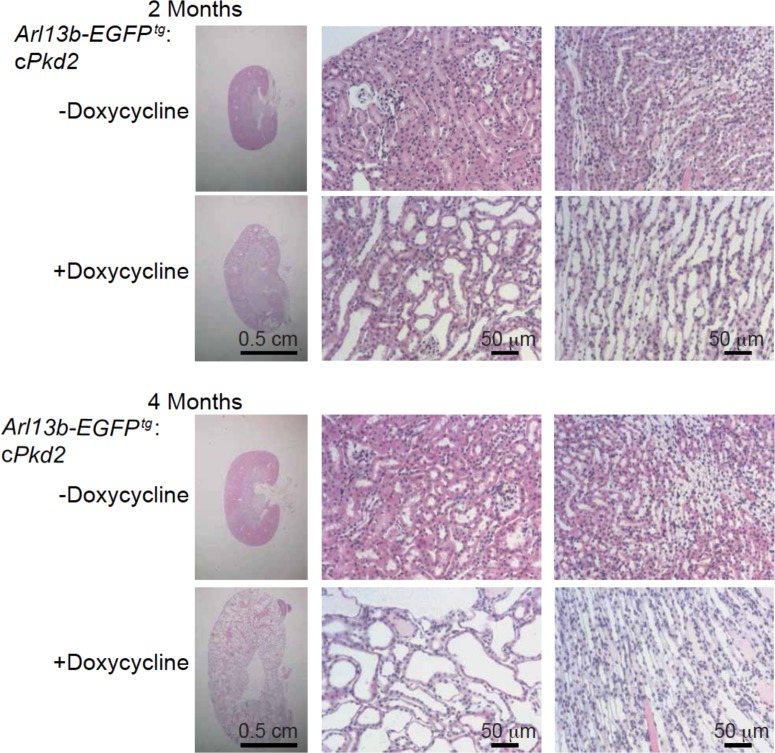
Progression of ADPKD after genetic ablation of *Pkd2*. Representative images of H and E staining of kidneys from *Arl13b-EGFP^tg^:cPkd2* mice. Sagittal sections of kidneys (left, scale bar, 0.5 cm) and higher magnification (middle and right, scale bar, 50 μm) are shown at the indicated times; three independent experiments.

**Figure 1—figure supplement 2.**
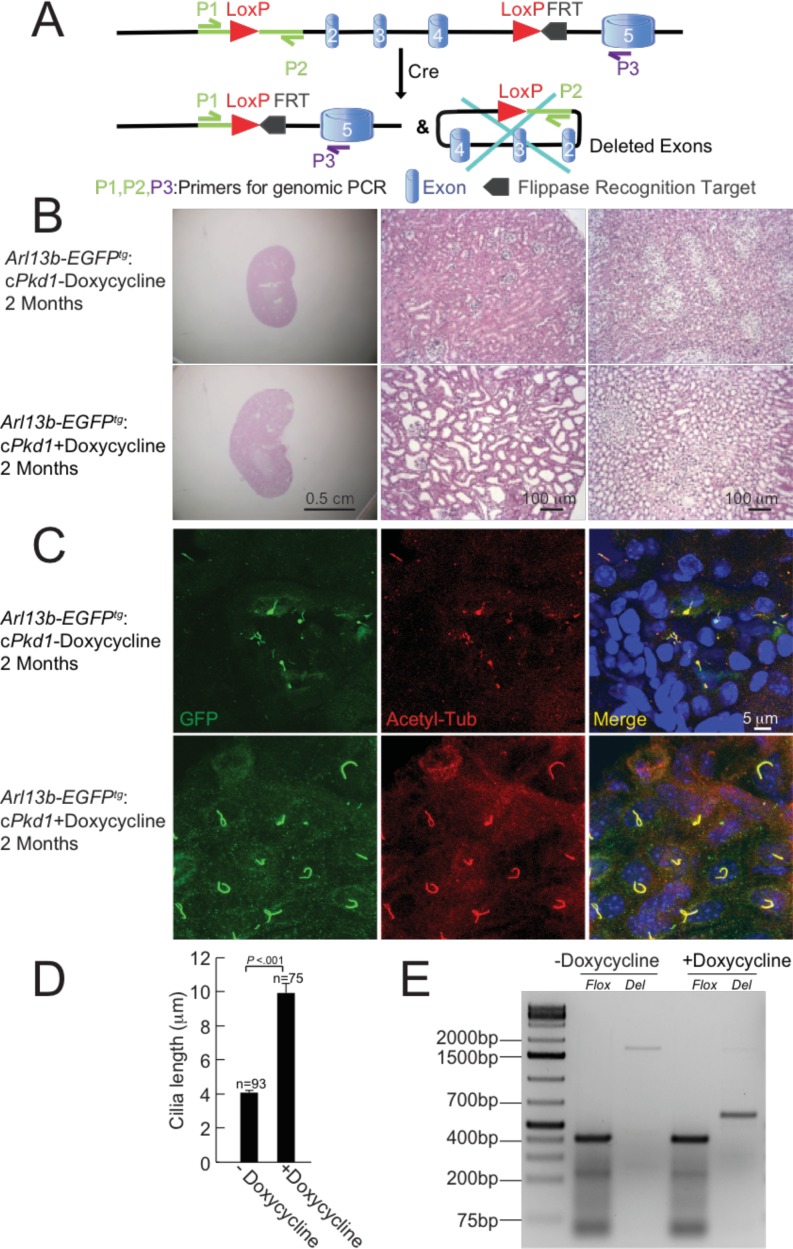
Progression of ADPKD after genetic ablation of *Pkd1*. (**A**) Schematic diagram of conditional inactivation of *Pkd1* in *Arl13b-EGFP^tg^:cPkd1 mice.*. (**B**) Representative images of H and E staining of kidneys from *Arl13b-EGFP^tg^:cPkd1* mice (2 months after doxycycline removal); three independent experiments. Sagittal sections of kidneys (left, scale bar, 0.5 cm) and higher magnification (middle and right, scale bar = 100 μm) are shown at the indicated times. (**C**) Representative kidney sections from *Arl13b-EGFP^tg^:cPkd1* mice (2 months after doxycycline removal) were immuno-labeled with antibodies against EGFP and acetylated tubulin; three independent experiments. Scale bar, 5 μm. (**D**) Cilia length increased with the progression of cyst formation from kidney sections of *Arl13b-EGFP^tg^:cPkd1* mice (2 months after doxycycline removal). (**E**) Genomic PCR for pIMCD cells isolated from *Arl13b-EGFP^tg^:cPkd1* mice (2 weeks after doxycycline removal); three independent experiments. The PCR product for the *floxed* allele,~400 bp; for *wt,*~200 bp; for the *Deletion* alleles (exons 2–4 of *Pkd1* deleted) of *Del,~*650 bp; for *non-Del,*~1650 bp. The weak band at ~1650 bp in the far right lane is the residual amount of intact *Pkd1*, which may be due to the contamination of non-tubule cells or tubule cells from S3 straight segment. The bands larger than 200 bp, both before and after doxycycline treatment, are non-specific bands (absent at shorter PCR extension times).

Immunoblots were performed from pIMCD cell lysates from 2 week and 2 month doxycycline-ablated *Arl13b-EGFP^tg^:cPkd2* or 2 week post-treatment of *Arl13b-EGFP^tg^:cPkd1* mice, indicating that the recombinase substantially reduced polycystin-2 or polycystin-1 protein expression (Figure 1B). Polycystin-2 expression from *Arl13b-EGFP^tg^:cPkd1* animals was unaffected by polycystin-1 ablation (Figure 1B). Consistent with previous reports from the *cPkd2 and cPkd1* strain, we observed kidney cyst formation in *Arl13b-EGFP^tg^:cPkd2* and *Arl13b-EGFP^tg^:cPkd1* mice (Figure 1—figure supplement 1, Figure 1—figure supplement 2B). The extent of cyst formation in these mice was quantified as the kidney-to-body weight ratio and cystic index (Figure 1C–F). Based on these measures, the cystic phenotype was progressive, as seen by comparing the post 2 month and 4 month treatment groups (Figure 1C–F, Figure 1—figure supplement 1, Figure 1—figure supplement 2B).

### Abnormal cilia in *Pkd1*- or *Pkd2*-ablated mice

**Figure 2.**
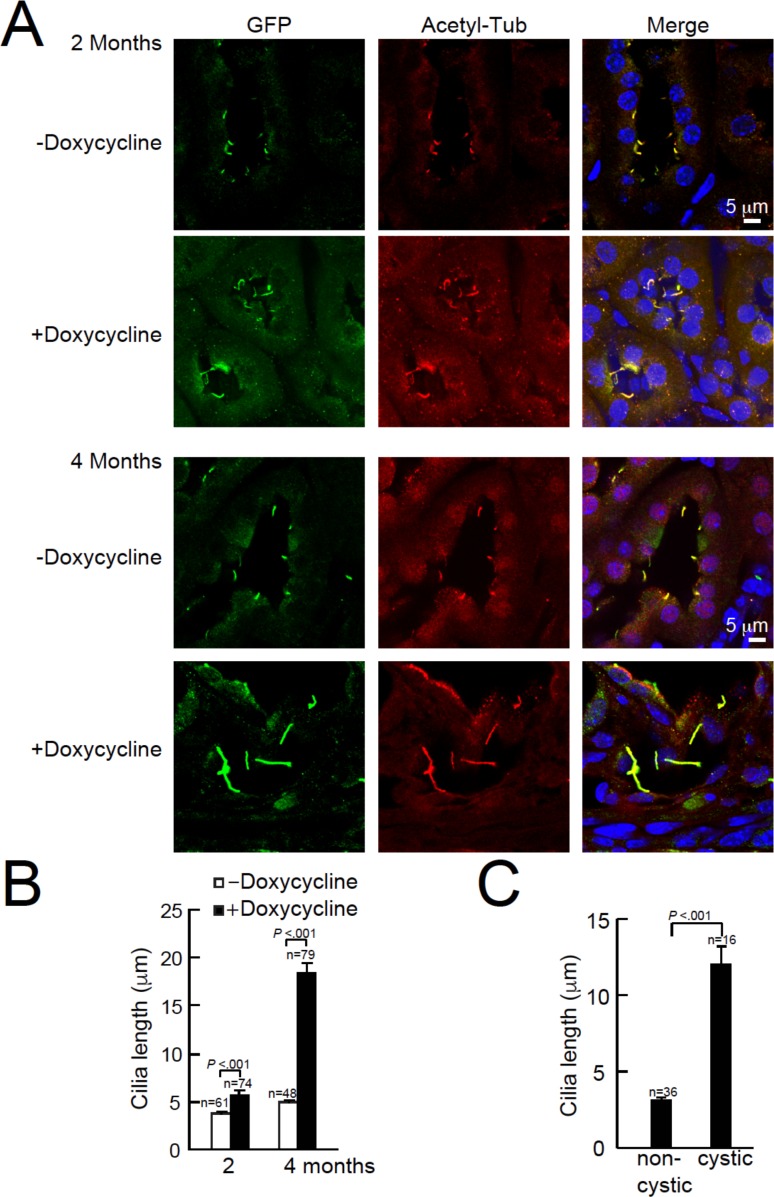
Cystic kidney cilia are abnormally long compared to unaffected tubules after genetic ablation of *Pkd2*. (**A**) Representative kidney sections from *Arl13b-EGFP^tg^:cPkd2* mice were immunolabeled with antibodies against EGFP and acetylated tubulin. Three independent experiments were performed. Scale bars, 5 μm. (**B**) Cilia length was measured with the progression of cyst formation from kidney of *Arl13b-EGFP^tg^:cPkd2* mice. (**C**) Cilia length was measured in cystic and non-cystic areas from the *Arl13b-EGFP^tg^:cPkd2* mice after 2 months of doxycycline removal.

Using confocal microscopy, we compared cilia morphology from kidneys of *Arl13b-EGFP^tg^:cPkd2* and *Arl13b-EGFP^tg^:cPkd1* mice treated with or without doxycycline (Figure 2A–C, Figure 1—figure supplement 2C–E). Here, we observed an ~3.2 fold increase in cilia length with the progression of ADPKD (5.7 ± 0.4 μm for 2 months and 18.4 ± 1.2 μm for 4 months post-treatment) with *Arl13b-EGFP^tg^:cPkd2* mice, whereas cilia length from control littermates did not differ substantially over the same time course (3.8 ± 0.16 μm and 4.5 ± 0.2 μm, respectively). Also, we found that cilia length from tubule cells lining cysts were ~4 times longer than from unaffected tubules from the same animals (Figure 2C) (12 ± 1.1 μm and 3.1 ± 0.2 μm, respectively). As for *Arl13b-EGFP^tg^:cPkd1* mice, we observed an ~2.4 fold increase in cilia length with the progression of ADPKD (4.1 ± 0.1 μm for control littermates and 9.9 ± 0.5 μm for 2 months post-treatment)(Figure 1—figure supplement 2D). These results demonstrate the neither polycystin-1 nor polycystin-2 expression is required for primary ciliogenesis from the tubule epithelium, but implies that polycystin-1 or polycystin-2 expression is somehow related to cilia length. Since aberrant cilia morphology was mostly found in cystic tissue epithelia compared to non-cystic tubules, ciliary polycystin-1 or polycystin-2 may regulate continuing renal tubular cell differentiation. However, it is unclear if irregular cilia morphology is a consequence or cause of cyst formation, and what function overexpression of *Arl13b-EGFP* in combination with polycystin-1 or polycystin-2 ablation may have in maintaining normal cilia length.

### Ciliary trafficking and ion channel activity of polycystin-2 are independent of polycystin-1

**Figure 3.**
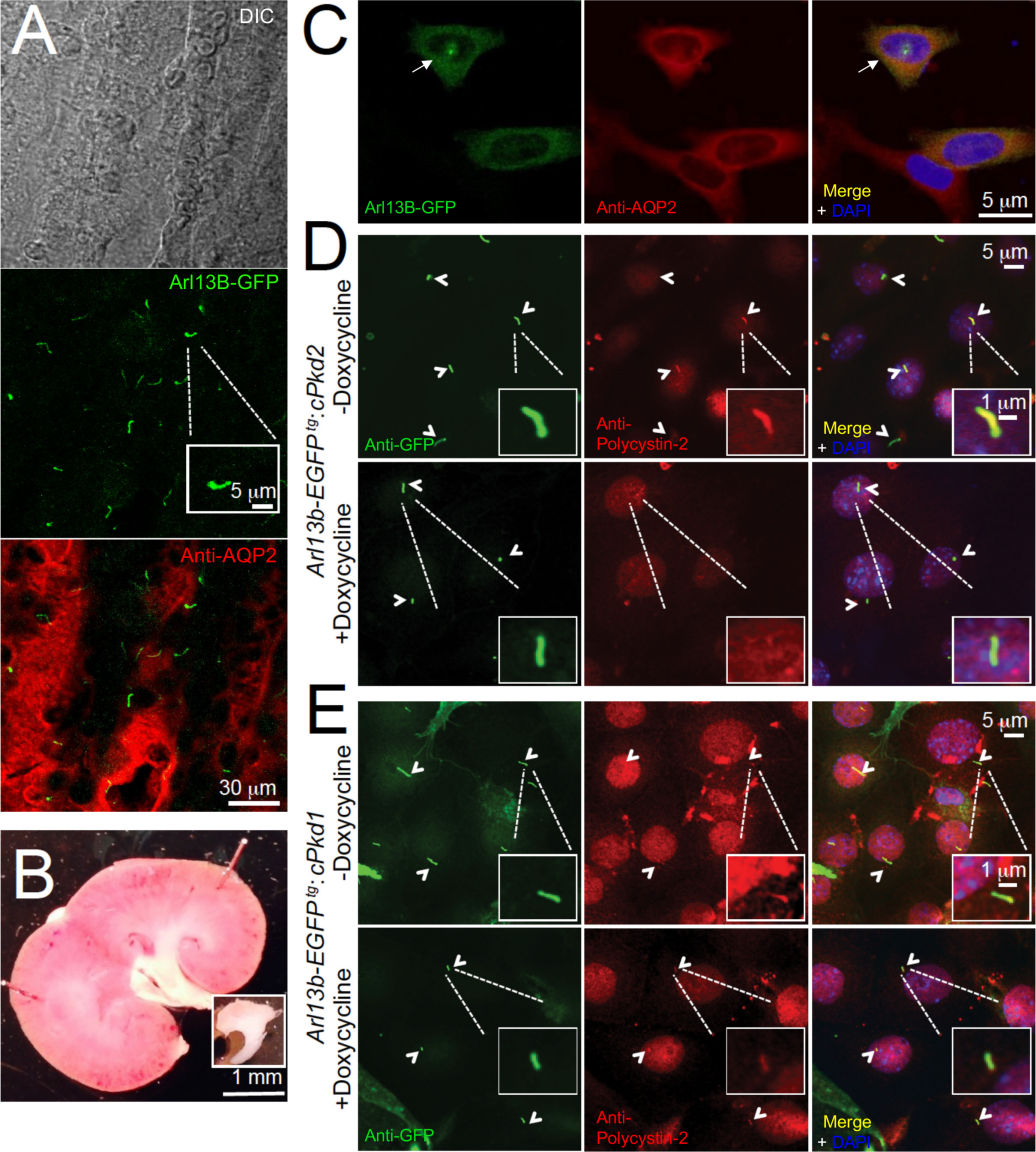
In situ and in vitro detection of ciliary polycystin-2 in *Arl13b-EGFP^tg^* pIMCD cells. (**A**) Confocal images from a 100 μm-thick fixed kidney slice: DIC image in grey; aquaporin 2 (kidney collecting duct epithelial cell epitope) labeled with Alexa-569 (red); Cilia *Arl13b-EGFP^tg^* (green). Three independent experiments were performed. Scale bar = 30 μm, inset image scale bar = 5 μm. (**B**) Sagittal section of 3-month-old mouse kidney with the inner medulla removed (bottom inset). Three independent experiments were performed. Scale bar = 1 mm. (**C**) Confocal images of fixed primary collecting duct epithelial cells after two days in culture, immunostained with anti-aquaporin 2 antibody in A). Three independent experiments were performed. Scale bar = 5 μm. (**D**) Immunofluorescence using anti-GFP (green) and anti-polycystin-2 (red) showing the loss of polycystin-2 in pIMCD cells isolated from kidney papillae of *Arl13b-EGFP^tg^:cPkd2* mice (2 weeks after doxycycline removal; three independent experiments; 5 mice were used for each group). Arrowheads point to primary cilia. Scale bar = 5 μM, inset image scale bar = 1 μm. (**E**) Immunofluorescence with anti-GFP (green) and anti-polycystin-2 (red), showing ciliary polycystin-2 in pIMCD cells isolated from kidney papillae of *Arl13b-EGFP^tg^:cPkd1* mice. Three independent experiments; 5 mice for each group. Arrowheads point to primary cilia. Scale bar = 5 μM, inset image scale bar = 1 μm.

Using animals from the same study design, we harvested pIMCD cells from 2-month-old *Arl13b-EGFP^tg^* mice, before cyst development. The cell membrane of the dissociated cells retained anti-aquaporin 2 antibody reactivity and Arl13B was found in the primary cilia of intact distal collecting ducts (Figure 3A–C). Using the validated antibody described in Figure 1, we confirmed the lack of ciliary polycystin-2 from cultured pIMCD cells from post-doxycycline-treated *Arl13b-EGFP^tg^:cPkd2* mice (Figure 3D). Importantly, the pIMCD cells isolated from post-doxycycline-treated *Arl13b-EGFP^tg^:cPkd1* animals retained their ciliary polycystin-2, suggesting that ciliary polycystin-2 trafficking does not require polycystin-1 (Figure 3E, Figure 1—figure supplement 2E).

**Figure 4.**
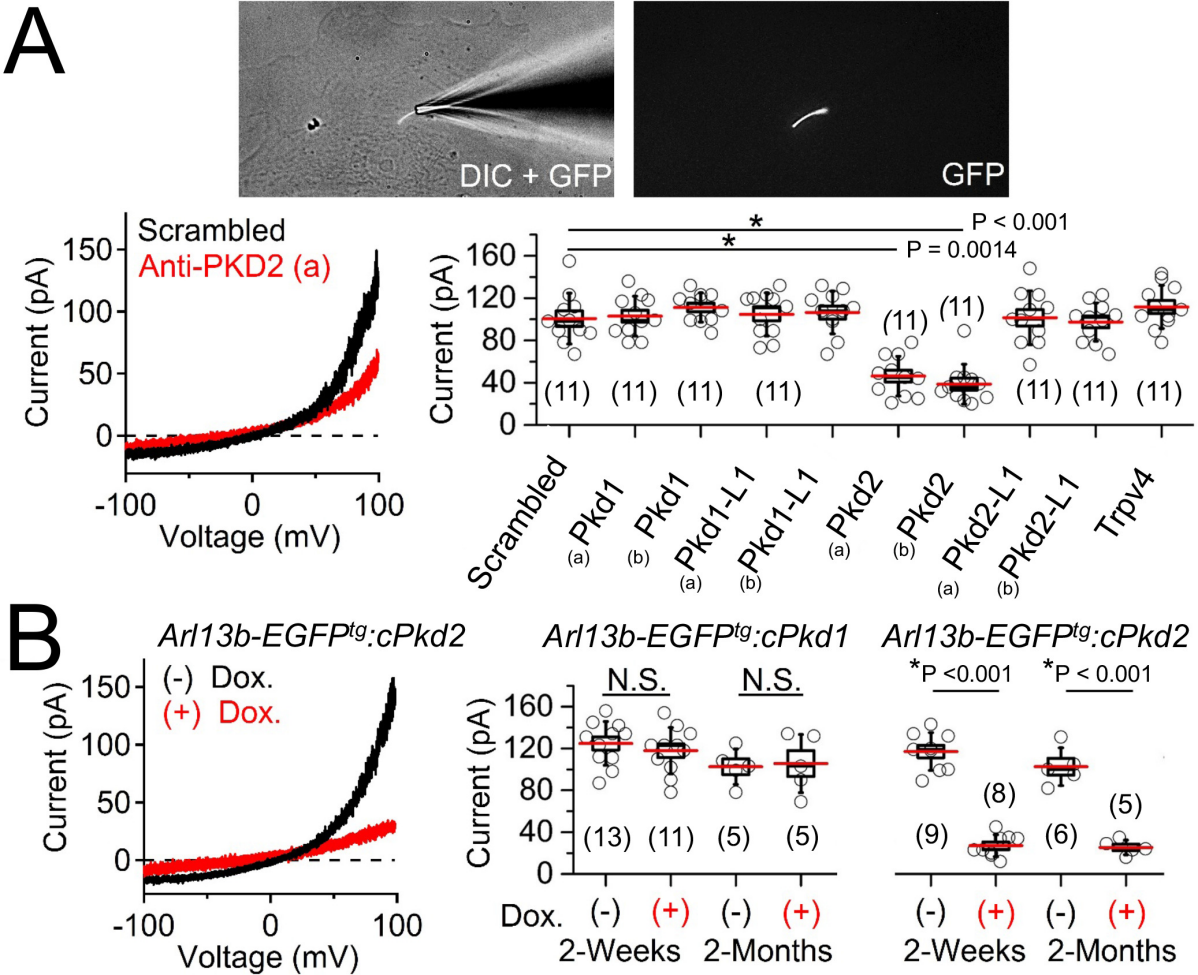
Polycystin-2 is required for the ciliary ion channel conductance of the primary inner medullary collecting duct epithelial cells (pIMCDs). (**A**) siRNA screen of potential I_cilia_ candidates. *Top*, light microscope image of a patched cilium. *Left*, example ciliary currents measured from cells treated with either scrambled siRNA or one targeted to polycystin-2. *Right*, box (mean ± S.E.M.) and whisker (mean ± S.D.) plots of cilia total outward current (+100 mV) measured 48–72 hr after double-siRNA treatment. *Pkd1*, *Pkd1-L1*, *Pkd2*, *and Pkd2-L1* mRNAs were targeted by two siRNAs specific for two different regions (**A, B**) of the target transcript (listed in Table 1). Averages are indicated by the red lines. Student’s *t*-test P values comparing treatment groups to scrambled siRNA. See Figure 4—source data 1-ciliary current amplitudes: siRNA screen of TRP proteins in cilia. (**B**) Conditional knockout of the whole-cilia current. *Left*, exemplar cilia currents from pIMCD epithelial cells isolated from conditional *Pkd2* knockout (*Arl13b-EGFP^tg^:cPkd2*) transgenic mice. *Right*, box and whisker plots comparing the total outward cilia current (+100 mV) from control littermates and doxycycline-treated animals (*Arl13b-EGFP^tg^:cPkd1* and *Arl13b-EGFP^tg^:cPkd2*). Number of cilia; italic numeral in parentheses for each group genotype and treatment group. Student’s *t*-test P values compare the outward cilia current from the untreated and doxycycline-treated animals. See Figure 4—source data 2- whole ciliary current amplitudes in *Pkd1* or *Pkd2*-knockout primary cells. 10.7554/eLife.33183.008Figure 4—source data 1.Ciliary current amplitudes: siRNA screen of TRP proteins in cilia.Ciliary outward currents (pA) were measured at 100mV after knockdown of *Pkd1, Pkd1-L1, Pkd2, Pkd2-L1* and *Trpv4*.Except for *Trpv4*, the mRNAs the genes tested were targeted by two siRNAs specific for two different regions, as labeled A and B. 10.7554/eLife.33183.009Figure 4—source data 2.Whole ciliary current amplitudes in *Pkd1* or *Pkd2*-knockout primary cells.Ciliary outward currents (pA) were measured at 100 mV from pIMCD epithelial cells isolated from doxycycline-treated and -untreated mice. Cells were tested 2 weeks and 2 months after removal of treatment (or controls) using *Arl13b-EGFP^tg^:cPkd1 and Arl13b-EGFP^tg^:cPkd2* transgenic mice

Next, we patch clamped pIMCD cells, in which the primary cilia could be visualized and expression of either polycystin-1 or polycystin-2 subunits of the putative ciliary ion channel complex could be conditionally controlled. Previously, we used the cilium patch method to identify the heteromeric polycystin 1-L1/polycystin 2-L1 channel in primary cilium of *Arl13b-EGFP^tg^* retinal pigmented epithelial cells (RPE) and mouse embryonic fibroblasts (MEF) (DeCaen et al., 2013). Also, we described, but did not identify, a large outward conductance channel (outward γ = 98 ± 2 pS) from the cilia of an immortalized IMCD-3 cell line, which has been characterized (outward γ = 96 pS) and subsequently identified as polycystin-2 by the Kleene group (Kleene and Kleene, 2017). Thus, we extended our cilia electrophysiology methods to test ciliary ion currents from pIMCD cells and determine if polycystin-1 and/or polycystin-2 are subunits of the ion channel. After establishing high resistance seals (>16 GΩ) at the tip of the cilia membrane (Video 1), we ruptured the cilium’s membrane and established ‘whole-cilium’ patch recording to observe an outwardly rectifying current (Figure 4A, Figure 4—source data 1-ciliary current amplitudes: siRNA screen of TRP proteins in cilia).

**Video 1.** Movie of the pIMCD *Arl13b* cilium patch configuration. The glass pipette patch electrode (*right*) is sealed onto a primary cilium above the pIMCD cell. The focal plane was moved along the z-axis (~9 µm) to visualize the cell and cilium. The electrode is moved along the y-axis while adjusting the focal plane to demonstrate that the patch electrode is sealed on the tip of the cilia membrane, not the cell membrane. The light source(s) are indicated in the lower left corner of the image: white light and 488 nm light to illuminate the specimen. Differential interference contrast (DIC) or fluorescent images were captured using a Hamamatsu Orca Flash CCD camera on an Olympus IX73 inverted microscope; 60x objective, 2x photomultiplier. Scale bar = 5 μm.

To determine the identity of this current, we treated cells with siRNA specific for members of the polycystin family and other localized putative ciliary ion channel subunits (Kleene and Kleene, 2017; Köttgen et al., 2008; Yoder et al., 2002). We observed 53% and 61% attenuation of whole-cilium current from cells treated with two independent siRNAs targeted to polycystin-2 (Figure 4A, Table 1, Figure 4—source data 1-ciliary current amplitudes: siRNA screen of TRP proteins in cilia). Importantly, we did not find any difference in currents when cells were treated with siRNAs targeting *Pkd1*, *Pkd1-1L1*, *Pkd2-L1,* and *Trpv4*, suggesting that none of these targets are essential subunits of the pIMCD ciliary current. To confirm these results, we measured ciliary current of pIMCD cells from *Arl13b-EGFP^tg^:cPkd2* mice at 2 weeks and 2 months after withdrawal of doxycycline treatment. As expected, the ciliary outwardly rectifying currents from the *Arl13b-EGFP^tg^:cPkd2* mice were reduced by 84% and 81% from 2 week and 2 month post-treatment groups compared to littermates not exposed to doxycycline (Figure 4B, Figure 4—source data 2-whole ciliary current amplitudes in *Pkd1* or *Pkd2*-knockout primary cells). These results demonstrate that doxycycline-induced TetO-cre ablation of *Pkd2* substantially reduces the pIMCD ciliary current. In contrast, cilia currents recorded from pIMCD cells isolated from doxycycline-treated *Arl13b-EGFP^tg^:cPkd1* mice do not have reduced current compared to cells from untreated animals from 2 week and 2 month post-treatment groups (Figure 4B, Figure 4—source data 2-whole ciliary current amplitudes in *Pkd1* or *Pkd2*-knockout primary cells). From this data, we conclude that polycystin-2 is a subunit of a major ion current in renal tubule epithelial cilia and that the absence of polycystin-1 expression does not substantially alter the net polycystin-2 current in the cilium.

**Table 1 .** siRNAs used to screen for ciliary ion channel genes.

siRNA gene target M, *Mus musculus*; h, *human*	ThermoFisher Silencer ID	siRNA location	% Knockdown efficiency
m*Pkd1* (a)	151949	999	75 ± 3
m*Pkd1* (b)	151951	7303	73 ± 3
m*Pkd1-L1* (a)	n398242	1075	76 ± 2
m*Pkd1-L1* (b)	n398244	806	72 ± 3
m*Pkd2* (a)	150154	1089	83 ± 2
m*Pkd2* (b)	63551	488	81 ± 2
m*Pkd2-L1* (a)	101318	350	84 ± 2
m*Pkd2-L1* (b)	101422	830	82 ± 2
m*Trpv4*	182203	778	80 ± 2
hPKD2 (a)	104317	1100	74 ± 2
hPKD2 (b)	143288	3069	71 ± 3

### Ciliary polycystin-2 preferentially conducts K^+^ and Na^+^ over Ca^2+^ ions

As discussed in the introduction, it is widely reported that calcium is a major charge carrier for polycystin-2 under physiological conditions. However, we find that the collecting duct epithelial cilia membrane is ~2.5 times more selective for potassium than sodium ions (relative permeability P_K_/P_Na_ = 2.4, Figure 5—figure supplement 1A). Here, the relative permeability was estimated by the measured change in reversal potential when sodium was replaced by each test cation (Table 2). To test calcium permeability, the 110 mM NaCl extracellular solution was replaced with equimolar CaCl_2_ (keeping 110 mM internal Na^+^) which negatively shifted the reversal potential (ΔE_rev_ = −57 mV, Table 2), indicating that permeation by calcium (P_Ca_/P_Na_ = 0.06) is barely different than presumably impermeant NMDG (P_NMDG_/P_Na_ = 0.04). We also tested the permeability of chloride (P_Cl-_) by substituting external Cl^-^ with the larger methane sulfonate while keeping [Na^+^] constant (110 mM NaCl vs. NaMES). Here, there was no difference in reversal potential when NaCl was exchanged for NaMES (ΔE_rev_ = −2 ± 3 mV, Table 2), demonstrating that P_Cl-_ is negligible in the pIMCD cilium membrane. These data also demonstrate that polycystin-2’s selectivity is strictly cationic and distinct from that previously reported for polycystin 1-L1/2-L1 recorded in the cilia of RPE and MEF cells, which was ~6 x more selective for Ca^2+^ over Na^+^ and K^+^ (DeCaen et al., 2013).

**Table 2.** Transmembrane reversal potentials (*E*_rev_) measured from pIMCD and HEK-293 PKD2-GFP cilia.

External solution	pIMCD, whole-cilium		HEK-293 PKD2-GFP, whole-cilium	
	*ΔE*_rev_ (mean ± S.D.)	P_x_/P_Na_ (mean ± S.D.)	*ΔE*_rev_ (mean ± S.D.)	P_x_/P_Na_ (mean ± S.D.)
NaCl	0	1	0	1
NaMES	−2 ± 3 mV	0.96	Not tested	-
KCl	26 ± 3 mV	2.4 ± 0.3	23 ± 4 mV	2.4 ± 0.4
CaCl_2_	−49 ± 4 mV	0.06 ± 0.04	−48 ± 3 mV	0.09 ± 0.04
NMDG	−57 ± 4 mV	0.04 ± 0.02	−58 ± 4 mV	0.04 ± 0.02

**Figure 5.**
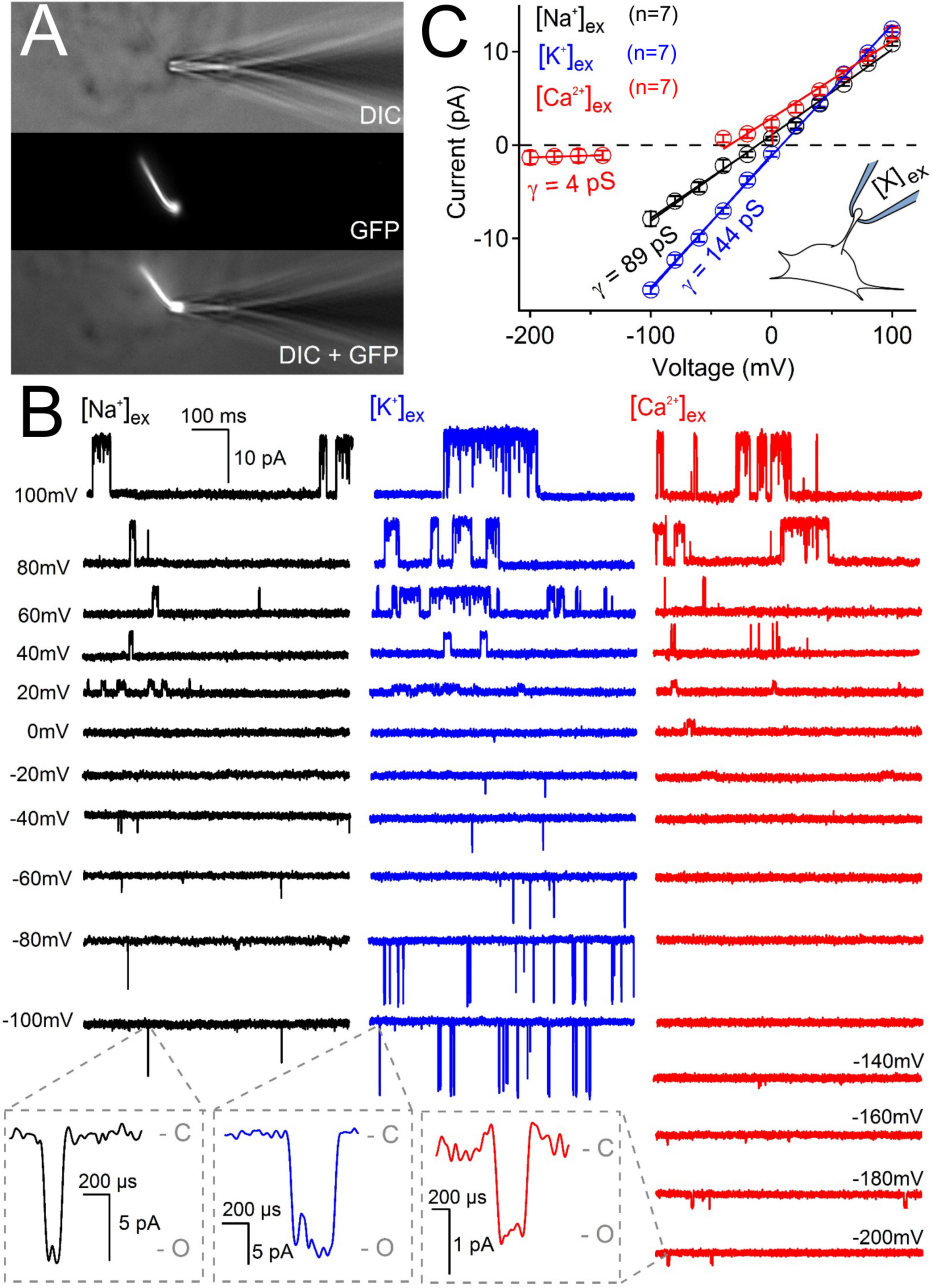
Ciliary polycystin-2 single channel currents conducted by sodium, potassium and calcium ions. (**A**) Image of a cilia patched without breaking into the cilioplasm (‘on-cilium’ configuration). (**B**) Exemplar currents recorded with the indicated cation (110 mM) in the patch electrode. Expanded time scales in the grey boxes show that inward single channel currents are brief, often opening (**O**) and closing (**C**) within 1 ms. (**C**) Average single channel current amplitudes. Conductance (γ) estimated by fitting the average single channel currents to a linear equation. Note that the inward single channel events are small (~0.8 pA at −200 mV) when Ca^2+^ is used as the charge carrier in the pipette (inset, patch diagram); note outward currents are much larger. Outward conductances of 90 pS, 99 pS and 117 pS, and inward conductances of 4 pS, 89 pS, and 144 pS were measured when the pipette contained Ca^2+^, Na^+^ and K^+^, respectively. *Inset*, a cartoon of the ‘on-cilium’ patch configuration where cations within the patch electrode ([X^+^]) are exclusively capable of conducting inward currents.

We tested the effect of changing external calcium ([Ca^2+^] _ex_) while maintaining a constant level of Na^+^ (100 mM) on the magnitude on the inward ciliary current (Figure 5—figure supplement 1B). Here we observed that the inward current, presumably carried by Na^+^, was antagonized by [Ca^2+^] _ex_ (IC_50_ = 17 mM). This appears to be a consistent feature of polycystin-2 and mutated forms of the polycystin-2 channels when recorded from oocytes and reconstitution preparations (Arif Pavel et al., 2016; Cai et al., 2004; Koulen et al., 2002; Vassilev et al., 2001). To validate our findings of ciliary relative permeability, we also compared the single channel conductance of inward Na^+^, K^+^, and Ca^2+^ when they were exclusively present in the pipette (cilium-attached configuration). Of the three ions tested, K^+^ conducted through ciliary polycystin-2 channels with the greatest inward conductance (γ_K_ = 144 ± 6 pS), followed by sodium (γ_Na_ = 89 ± 4 pS) and calcium (γ_Ca_ = 4 ± 2 pS) (Figure 5). The inward Ca^2+^ single channel currents were only observed under high electrical driving forces – when the ciliary membrane was hyperpolarized more negative than −140 mV (Figure 5B,C). Note that the outward conductance for all three conditions ranged between 90–117 pS, suggesting that the outward conductance is likely a mixture of Na^+^ and K^+^ exiting the cilium. The inward single channel open events were brief, usually lasting less than 0.5 ms (I_Na_ open time 0.4 ± 0.2 ms at −100 mV), whereas those measured at positive potentials opened for 190 times longer (I_Na_ open time 76 ± 29 ms at 100 mV). Importantly, the inward and outward conductance were absent from pIMCD cilia patches measured from doxycycline-treated *Arl13b-EGFP^tg^:cPkd2* animals when Ca^2+^ was used in the pipette (Figure 5—figure supplement 2A–C), demonstrating that the conductance (both inward and outward) is dependent on *Pkd2* expression. When we compare the extrapolated ΔE_rev_ (−61 mV) from single channel Ca^2+^ and monovalent currents, we observe that P_Ca_/P_mono_ = 0.04 when we assume that [Ca_cilia_] is high (580 nM) (Delling et al., 2013) and the cumulative ciliary monovalent concentration (155 mM) is similar to the cytosol (Figure 5—figure supplement 2D). Thus, the relative permeability of ion conductance dependent on polycystin-2 expression agrees with the relative permeability measured from the cilia membrane and the polycystin-2 single channels currents. We conclude that the major polycystin-2 conductance is monovalent, with relatively little inward Ca^2+^ flux.

**Figure 5—figure supplement 1.**
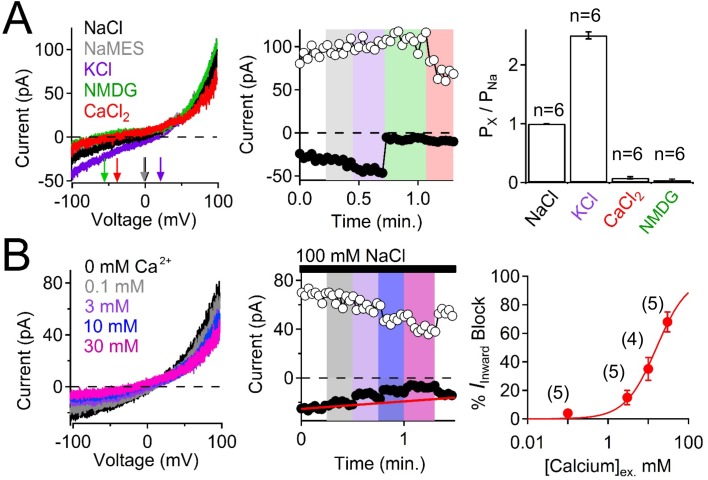
The pIMCD ciliary polycystin-2 cilia membrane is highly permeable to K^+^ and Na^+^. (**A**) Selectivity of the ciliary polycystin-2 current. *Left*, Example cilia current traces measured under different external cationic bi-ionic conditions. *Middle*, Time course of the magnitudes of inward (−100 mV, black circles) and outward (100 mV, white circles) currents with changes in the external solution. *Right*, Relative permeability of cations to Na^+^ through the polycystin-2 channel. (**B**) Attenuation of inward polycystin-2 sodium current in response to external calcium ([Ca]_ex_). *Left*, the effect of external calcium on the outwardly rectifying polycystin-2 cilia current measured in symmetrical sodium. *Middle*, corresponding time course of the magnitudes of inward (−100 mV, black circles) and outward (100 mV, white circles) currents upon changes in the extracellular solution. *Right*, relationship between external calcium concentration and percent block of the inward sodium current (*I_inward_*) fit to the Hill equation. Number of cilia tested are indicated by the italicized number in parentheses.

**Figure 5—figure supplement 2.**
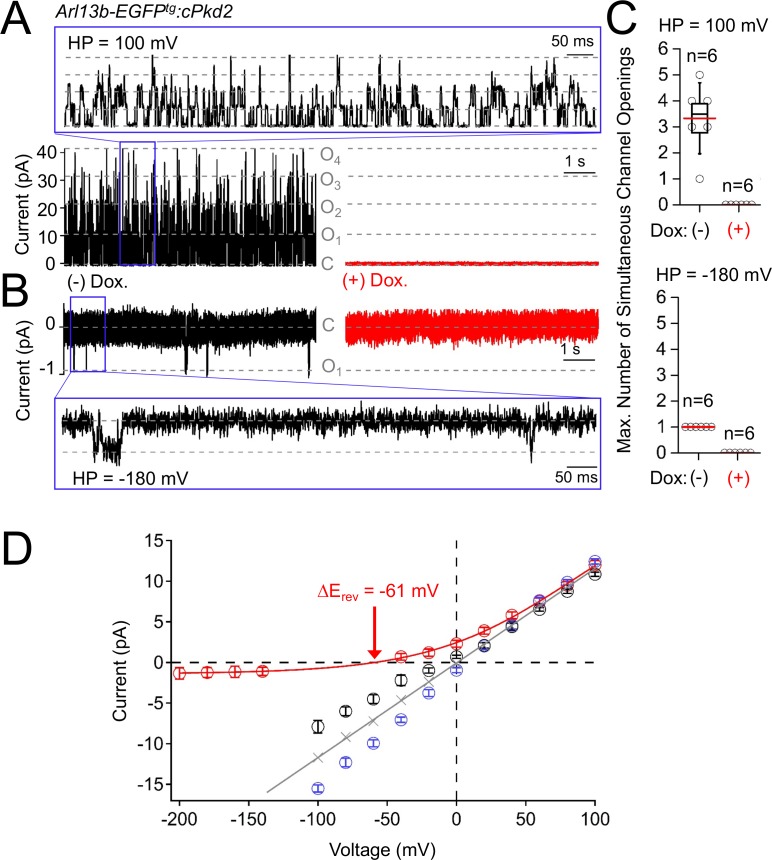
pIMCD single channel events are dependent on polycystin-2 expression. (**A**) Outward and B) inward single channel ciliary currents recorded from pIMCD cells harvested from doxycycline treated (red traces) and untreated (black traces) *Arl13b- EGFP^tg^:cPkd2* transgenic mice. Pipette electrodes contained 100 mM CaCl_2_ and the ciliary membrane potentials were held at 100 and −100 mV for up to 60 s (exemplars of 10 s of recording are shown) to record the number of simultaneous open events (grey, (**O_1_–O_4_**). Expanded time scales are shown in the blue boxes. (**C**) Box and whisker plots comparing the maximum number of simultaneous open channels observed from the two treatment groups. Six cilia were tested for each treatment group. (**D**) Derived from Figure 5, the reversal potential for average single channel calcium currents. The change in reversal potential ΔE_rev_ (−61 mV) was calculated by subtracting the E_Ca_ (- 59 mV) from the monovalent (E_mono_; 2 mV) reversal potentials, shown in grey.

### Ciliary polycystin-2 is sensitized by intraciliary free calcium

**Figure 6.**
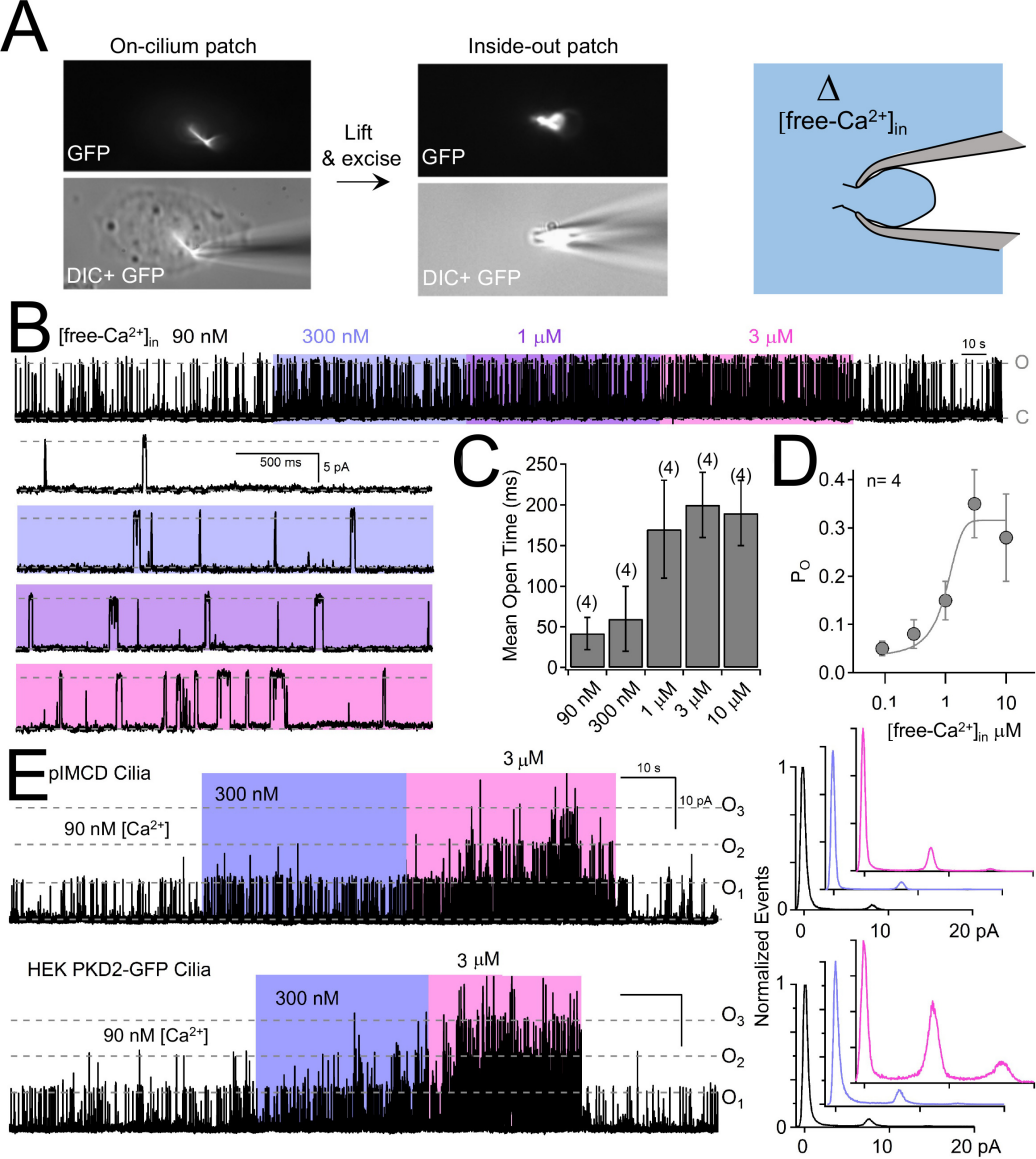
Internal [free-Ca^2+^] potentiates polycystin-2 channels. (**A**) Images recorded while establishing an inside-out cilium patch. *Left*, a high-resistance seal is formed on the cilium; *Middle*, the electrode is then lifted, ripping the cilium from the cell body (see Materials and methods); *Right*, cartoon depicting the inside of the cilium exposed to bath saline (blue) in which [Ca^2+^] can be adjusted. (**B**) pIMCD polycystin-2 single channel events recorded in the inside-out configuration. The membrane potential was held at +100 mV in symmetrical [Na^+^] while the internal [Ca^2+^] was altered for 40–60 s intervals. (**C**) The average open time duration relative to internal [Ca^2+^] (mean ± S.D.). (**D**) Average open probability as a function of internal [Ca^2+^], fit to the Hill equation (described in Materials and methods, mean ± S.D.). (**E**) *Left*, exemplar inside-out cilium patch records from pIMCD cilia and HEK-293 cilia with heterologously expressed polycystin-2 channels. *Right*, current histograms capturing multiple open channel events under high internal [Ca^2+^] conditions. Currents were normalized to the closed (0 pA) state amplitude for each internal [Ca^2+^].

Since intracellular calcium has been reported to sensitize polycystin-2 from the ER and cilia of cell lines (Cai et al., 2004; Kleene and Kleene, 2017) and the polycystin-like channel (also called polycystin -L or polycystin 2-L1) (DeCaen et al., 2016), we examined this property in pIMCD ciliary polycystin-2 channels. Inside-out cilia membrane single channel activity can be compared to varying levels of intraciliary free calcium ([free Ca^2+^]_in_) (Figure 6A). Most commonly, inside-out ciliary patches exhibited at least 3–4 active polycystin-2 channels, but some had only one polycystin-2 channel present (Figure 6B). We used these rare patches to determine that 3 μM [free Ca^2+^]_in_ enhanced the open probability of the polycystin-2 current ~10 times (increasing P_o_ from 0.034 ± 0.02 to 0.36 ± 0.07) and the mean open time ~6 times (increasing from 37 ± 26 ms to 215 ± 40 ms) compared to standard cytoplasmic concentrations of 90 nM [free Ca^2+^]_in_ (Figure 6B–D). The half maximal enhancement of IMCD polycystin-2 open time was 1.3 μM. Previously, internal ciliary calcium was shown to negatively shift the voltage dependence of polycystin-2 channel activation in IMCD-3 cell lines (Kleene and Kleene, 2017). To determine internal calcium’s effect of on polycystin-2 inward current at the cilia’s resting membrane (RMP_cilia_ = −18 mV) (Delling et al., 2013), we compared polycystin-2 single channels as we increased [free Ca^2+^]_in_. First, we measured single currents activated by voltage ramps (−100 to 100 mV) and subtracted the remaining ohmic current after channel inactivation (Figure 6—figure supplement 1A). At 600 nM [free Ca^2+^]_in_, the polycystin-2 channel typically remains closed at negative potentials (Figure 6—figure supplement 1B). However, when [free Ca^2+^]_in_ was increased to 30 μM, the open events were more frequent, which increased in the total inward current ~10 fold (summing the single channel events; −13 pA to −122 pA at RMP_cilia_, Figure 6—figure supplement 1B,C). Similar to the observations made from the cilia current recorded from IMCD-3 cilia, increasing [free Ca^2+^]_in _50-fold substantially shifts the voltage dependence of activation; Figure 6—figure supplement 1D). We also observed a ~ 10 x increase in the normalized conductance at RMP_cilia_(0.05 to 0.49), which was also observed at more negative potentials. In summary, ciliary calcium enhances both the inward and outward current, as is evident in the G/G_max_ vs. voltage relation.

**Figure 6—figure supplement 1.**
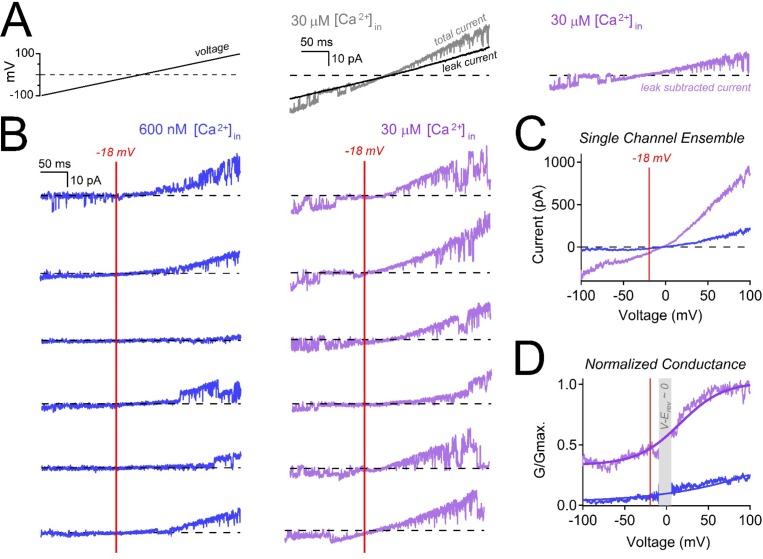
Internal calcium hyperpolarizes polycystin-2’s voltage dependence. (**A**) Inside-out cilia current traces captured before (left) and after leak subtraction (right). After 1–2 min in 30 μM internal calcium, the polycystin-2 currents inactivate, leaving only the leak current remaining. We averaged the leak current from five ramps (black) and subtracted it from the total current (grey), yielding the leak subtracted single channel currents (purple). (**B**) Six exemplar leak-subtracted single channel currents measured in 600 nM (left) and 30 μM (middle) internal calcium. For reference, the red line at −18 mV indicates the average expected cilia resting membrane potential. (**C**) Ensemble average of single channel currents recorded from 40 ramps. (**D**) Polycystin-2 conductance normalized to the maximum conductance measured in 30 μM internal calcium (*top trace, purple*). Conductance-voltage relationships were fit to a Boltzmann equation. Grey bar indicates where V-E_rev_ approaches 0 mV. The V_1/2_ in 600 nM and 30 μM internal calcium were 158 mV and 15 mV, respectively.

### Polycystin-2 functions as a channel in primary cilia, but not in the plasma membrane

**Figure 7.**
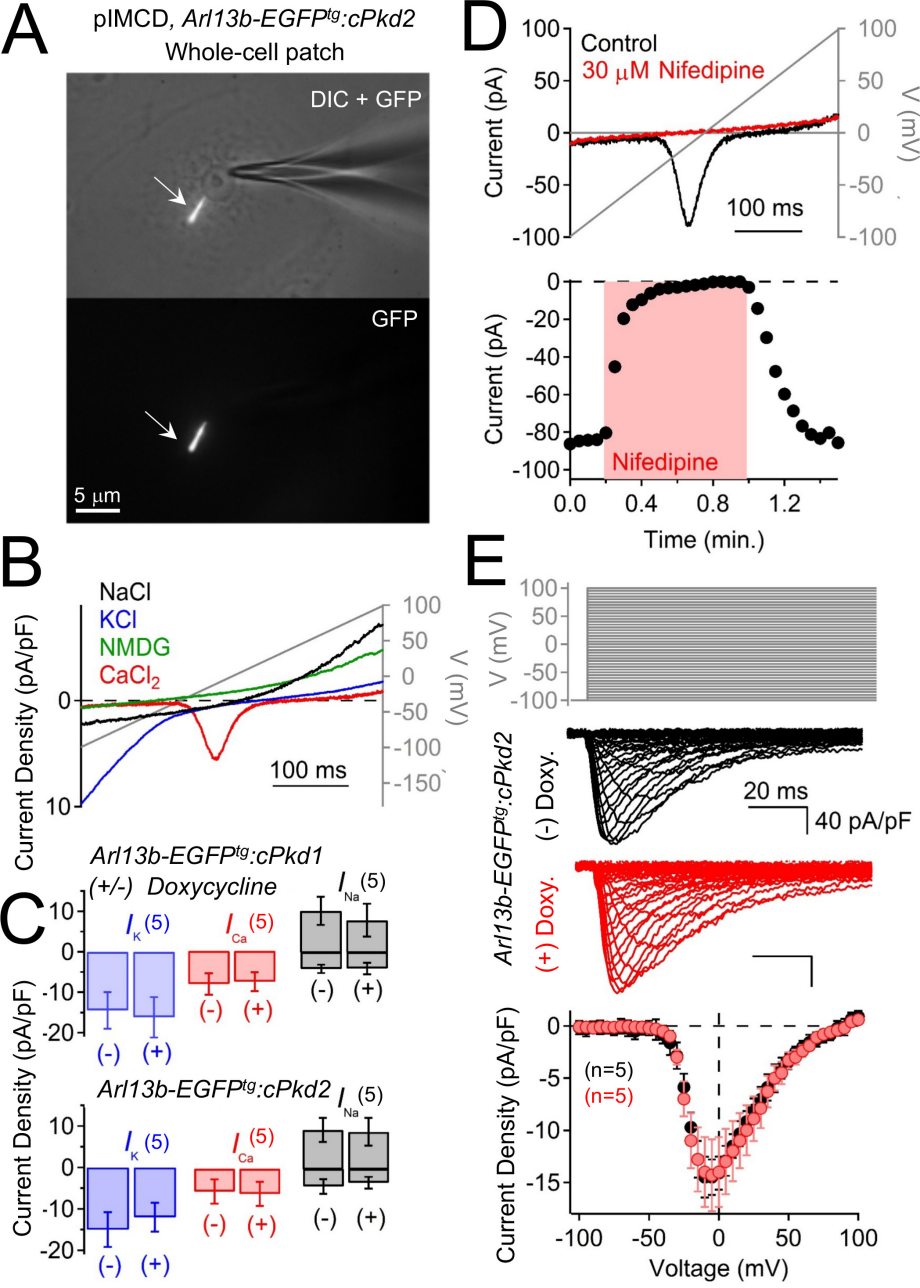
Polycystin-2 does not generate a significant current in the plasma membrane of pIMCD cells. (**A**) Images of a ciliated pIMCD epithelial cell patch-clamped on *the plasma membrane* (whole-cell mode). Scale bar = 5 μM. (**B**) Example plasma membrane ionic currents activated by a voltage ramp (grey line) while changing the extracellular saline conditions. (**C**) Average plasma membrane current density of *Arl13b-EGFP^tg^:cPkd2,* where animals treated with doxycycline was compared to untreated animals. Note that the average magnitudes of the plasma membrane currents were not significantly altered (mean ± S.E.M.). (**A**). (**D**) Pharmacological blockade of the voltage-gated calcium channel in the plasma membrane; three independent experiments. *Top*, exemplar calcium currents blocked by nifedipine. *Bottom,* time course of block and recovery of Ca_V_ currents. (**E**) Conditional polycystin-2 knockout does not alter the steady state voltage-gated calcium currents measured from the plasma membrane. *Top*, voltage protocol used to activate the calcium currents. *Middle*, exemplar leak-subtracted voltage-gated calcium currents from doxycycline-treated (red) and –untreated (black) *Arl13b-EGFP^tg^:cPkd2* animals. Resulting plasma membrane average Ca_V_ densities compared from doxycycline-treated and control littermates.

The above results confirm the location of functional ciliary polycystin-2 channels. Since the single channel recordings are made from the tips of cilia, polycystin-2 is present on the cilia membrane itself, not just the cilium/plasma membrane junction. However, native polycystin-2 channel are reportedly constitutively active in the plasma membrane of immortalized cell lines derived from kidney epithelial cells (mIMCD-3 and Madin-Darby canine kidney, MDCK, cells) (Luo et al., 2003). To test this possibility, we voltage clamped the plasma membrane of pIMCD cells harvested from *Arl13b-EGFP^tg^:cPkd2* mice (Figure 7A; Video 2). Here, we typically observed an outwardly-rectifying Na^+^-permeant current, an inwardly-rectifying K^+^ current, and an apparent voltage-gated Ca^2+^ current (Figure 7B). However, when polycystin-2 was conditionally reduced, there was no difference in plasma membrane currents densities (Figure 7C). Polycystin-2 function has been implicated in calcium transients originating in the endoplasmic reticulum (ER), plasma membrane, and cilium (Ma et al., 2005; Nauli et al., 2003; Qian et al., 2003). However, the plasma membrane current-voltage relationship, inactivation kinetics and pharmacology are typical of L-type calcium currents and did not change when polycystin-2 was reduced in these cells (Figure 7D,E). Thus, our findings suggest that polycystin-2 does not constitute a significant portion of the plasma membrane current found in primary collecting duct epithelial cells and does not alter the native voltage-dependent calcium current.

**Figure 7—figure supplement 1.**
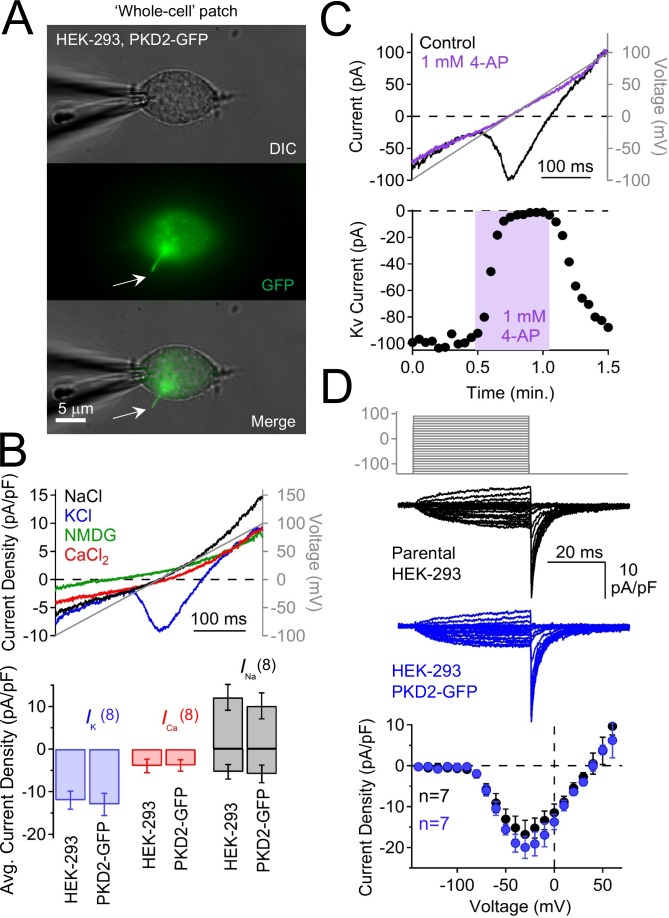
Characterization of plasma membrane currents measured from HEK-293 cells stably expressing polycystin-2-GFP. (**A**) Image of a voltage-clamped HEK-293 cell stably expressing polycystin-2-GFP. Note that the GFP signal is present in the primary cilium (arrow) and that the patch electrode is sealed onto the plasma membrane. Scale bar = 5 μM. (**B**) *Top*, resulting currents activated by a voltage ramp (grey line) under the indicated cationic conditions (in mM). Bottom, average current density for HEK-293 cells and those stably overexpressing polycystin-2-GFP. The current densities do not differ between the two cell types. (**C**) Pharmacological blockade of the voltage-gated potassium current (K_v_) in the HEK-293 plasma membrane; three independent experiments. *Top*, Example K_v_ currents recorded before and after 4-aminopyridine (4-AP) exposure. *Bottom*, time course of block and recovery of K_v_ currents. (**D**) Overexpressed polycystin-2 does not increase K_v_ current. Steady-state K_v_ currents measured from the plasma membrane. *Top*, voltage protocol used to activate K_v_ currents. *Middle*, exemplar leak-subtracted K_v_ currents from HEK-293 and those stably expressing polycystin-2-GFP. *Bottom*, K_v_ current density of HEK-293 and HEK-293 polycystin-2-GFP cells. Note that K_v_ current measured from HEK-293 cells is not different in HEK-293 cells overexpressing polycystin-2-GFP.

**Figure 7—figure supplement 2.**
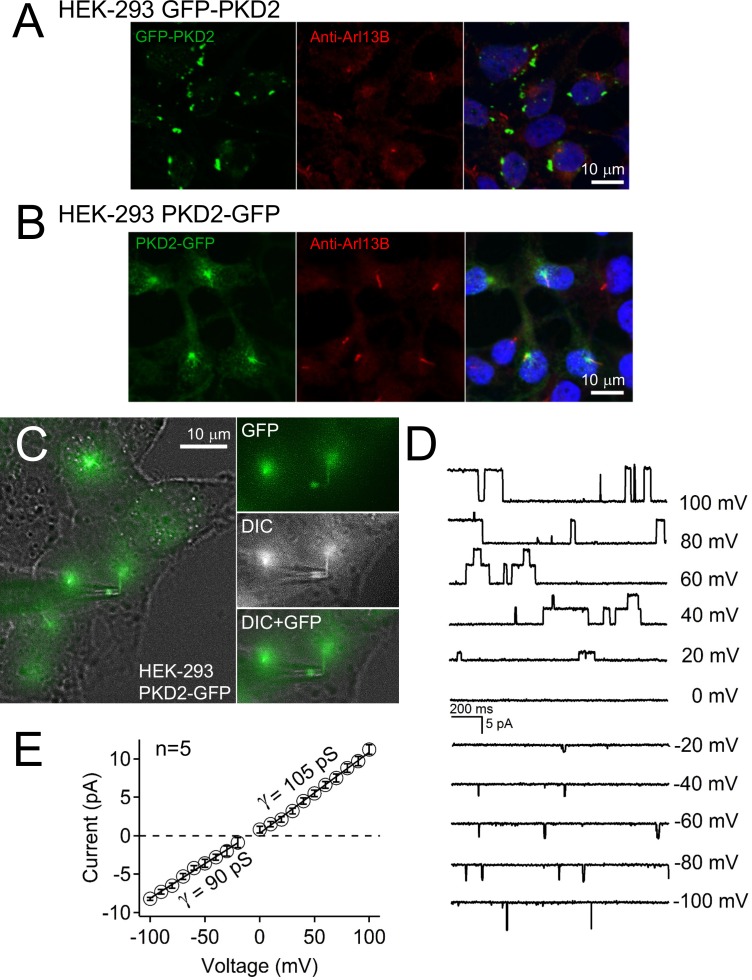
Single channel events recorded from cilium of polycystin-2-GFP overexpressing HEK-293 cells. (**A**) Stably expressing GFP-polycystin-2 HEK-293 cells were fixed, immunolabeled with rabbit anti-Arl13B primary antibody, and detected by a secondary conjugated anti-rabbit Alexa 569 antibody (red). Nuclei were stained with Hoechst 33342; three independent experiments. Scale bars = 10 μm. (**B**) Stably-expressing polycystin-2-GFP HEK-293 cells were fixed, immunolabeled with rabbit anti-Arl13B primary antibody, and detected by a secondary conjugated anti-rabbit Alexa 569 antibody (red). Nuclei were stained with Hoechst 33342; three independent experiments. Note that the Arl13B and polycystin-2-GFP signal colocalized to the cilium. (**C**) Live-cell image of an HEK-293 polycystin-2-GFP-labelled cilium patched in the on-cilium configuration. Note: the cilium’s tip is engulfed in the lumen of the electrode. Scale bars = 10 μM. (**D**) Exemplar single-channel currents recorded with a 100 mM NaCl-containing patch electrode. (**E**) Resulting outward and inward conductances (γ) estimated by fitting the single-channel currents to a linear equation.

**Figure 7—figure supplement 3.**
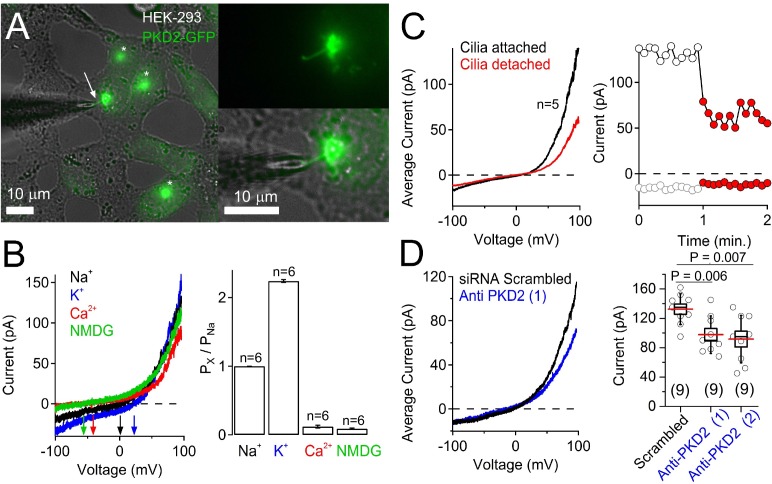
Overexpressed polycystin-2 forms an ion channel in the HEK-293 cilium. (**A**) Image of a voltage-clamped cilium from HEK-293 cells stably expressing polycystin-2-GFP. Note that the GFP signal and/or cilia is not always present on HEK-293 cells. Scale bar = 10 μM. (**B**) The selectivity of the ciliary current measured from HEK-293 polycystin-2-GFP cells. *Left*, Example ciliary current traces measured under different extracellular cationic bi-ionic conditions. *Right*, relative permeability of cations to Na^+^ through the polycystin-2 channel. (**C**) The majority of the ciliary current is conducted through the ciliary membrane. *Right*, Average current traces from five voltage ramps acquired before and after the cilia is disconnected from the cell membrane. *Left*, time course of peak current amplitudes before (white circles) and after (red circles) the cilia is separated from the cell. (**D**) *Left*, average current traces from cells treated with scrambled or polycystin-2-mRNA-targeted siRNAs. *Right*, Box (mean ± S.E.M.) and whisker (mean ± S.D.) plots of ciliary total outward (+100 mV) current amplitude. Averages are indicated by the red lines. See Figure 7—figure supplement 3—source data 1- ciliary current amplitudes of cells treated with *PKD2*-mRNA-targeted siRNAs . The GFP signal was noticeably reduced in antisense-treated polycystin-2 cells. Few cilia had as similarly intense fluorescence as the patched cilia (data not shown). 10.7554/eLife.33183.022Figure 7—figure supplement 3—source data 1.Ciliary current amplitudes of cells treated with *PKD2*-mRNA-targeted siRNAs.Ciliary outward currents (pA) were measured at 100 mV from *PKD2*-GFP stably-expressing HEK-293 cells treated with two *PKD2*-mRNA-targeted siRNAs.

**Video 2.** Visualization of a plasma membrane patch from a ciliated pIMCD *Arl13b*-GFP cell. Same method as Video 1. The cell membrane is being sealed by the patch electrode (*right*) and the primary cilium (*left*) is not in contact with the electrode. Scale bar = 10 µm.

### Polycystin-2 is a ciliary channel but is not constitutively active on the plasma membrane in HEK-293 cells

Previously, our attempts to record heterologously-expressed polycystin-2 currents from the plasma membrane using transient transfection from multiple cell lines were unsuccessful. Thus, we generated two HEK-293 stable cell lines which overexpressed either human *PKD2* with the C-terminus (*PKD2-GFP*) or N-terminus tagged with GFP (*GFP-PKD2*). In fixed preparations and live cells viewed with confocal and standard fluorescence microscopy, GFP-polycystin-2 was intracellular, while polycystin-2-GFP also localized to primary cilia (Figure 7—figure supplement 1A; Figure 7—figure supplement 2A,B; Video 3). The ciliary localization of polycystin-2-GFP in HEK-293 cells was confirmed in super-resolution images in which GFP co-localized with the known ciliary proteins, acetylated tubulin, and adenylyl cyclase (AC3) (Video 4; Video 5, respectively). However, we could not rule out polycystin-2 functioning in the plasma membrane of *PKD2-GFP* cells by fluorescence alone. To address this, we voltage clamped the HEK-293 *PKD2-GFP* whole-cell ion currents and found no difference when compared to parental HEK-293 cells (Figure 7—figure supplement 1D, surface area of cilia <2% of total plasma membrane). Since native ciliary pIMCD polycystin-2 channels preferentially conduct K^+^, we compared the plasma membrane potassium current using steady-state voltage protocols. The resulting current-voltage relationship and block by 4-AP (4-aminopyridine, a K^+^ channel antagonist) suggests that the HEK-293 K^+^ current is conducted by native voltage-gated potassium channels (Figure 7—figure supplement 1C), commonly reported in these cells (Foley and Boccuzzi, 2010; Sands and Layton, 2014; Spandidos et al., 2008; Wilkes et al., 2017). Importantly, the kinetics and magnitude of the plasma membrane potassium current was not altered when *PKD2-GFP* was stably overexpressed (Figure 7—figure supplement 1D). Thus, overexpressed *PKD2-GFP* by itself does not appear to form a constitutively active or voltage-gated channel in the nonciliary plasma membrane.

**Video 3.** A plasma membrane patch established on a polycystin-2-GFP HEK-293 cell. Here the whole-cell configuration is formed by the patch electrode (*left*) on the cell body and the primary cilium (*lower left*) is not in contact with the electrode. The electrode is moved along the y-axis while adjusting the focal plane to demonstrate that the cell membrane is sealed on the patch electrode, not the cilia membrane. Scale bar = 5 μm.

**Video 4.** Ciliary co-localization of polycystin-2-GFP with immunolabeled acetylated tubulin. Paraformaldehyde-fixed HEK-293 cells stably expressing polycystin-2-GFP (*green*) were immunolabeled with anti-acetylated tubulin antibody (*red*). The images were captured using Structured Illumination Microscopy (SIM; Nikon N-SIM scope) and 3D images rendered using Imaris software (Oxford Instruments). Scale bar = 3 μm.

**Video 5.** Cilia co-localization of polycystin-2-GFP with immunolabeled adenylyl cyclase 3. Paraformaldehyde-fixed HEK-293 cells stably expressing polycystin-2-GFP (*green*) were immunolabeled with anti-adenylyl cyclase three antibody (*red*). The images were recorded using a Nikon N-SIM scope and 3D images rendered using Imaris software (Oxford Instruments). Scale bar = 3 μm.

In contrast to the whole-cell recordings above, when we patch clamped the ciliary membrane of the *PKD2-GFP* HEK-293 cells, a large outwardly rectifying single channel conductance (γ = 105 ± 4 pS) was observed (Figure 7—figure supplement 2C–E). The *PKD2-GFP* HEK-293 ciliary inward conductance (γ = 90 ± 3 pS) was similar to the native polycystin-2 channels found in pIMCD cells (γ = 89 ± 4 pS) when sodium was used as a charge carrier. In the whole-cilium configuration, the cilia membrane primarily conducted K^+^ and Na^+^ with little permeation by Ca^2+^ (Figure 7—figure supplement 3A,B). To determine the identity of the outward rectifying current, we tested two siRNAs targeted to the overexpressed human *PKD2* and observed a 29% and 32% decrease in outward current compared to scrambled controls (Figure 7—figure supplement 3D, Figure 7—figure supplement 3D—source data 1-ciliary current amplitudes of cells treated with *PKD2*-mRNA-targeted siRNAs). Endogenous human polycystin-2 channels may be present in the cilia of HEK-293 cells and may contribute to some of the measured current. Future work genetically removing endogenous *PKD2* expression in HEK-293 cells will be necessary to study polycystin-2 channels in isolation and the impact of ADPKD-causing variants without the possibility of contaminating endogenous channels. To determine the localization of the channels mediating this current, we removed the cilia from the cell body by obtaining excised whole-cilium configuration recordings (Figure 7—figure supplement 3C). Excised patches from *PKD2-GFP* overexpressing HEK-293 cells retained an average of 44 ± 21% of the whole-cilium current. This percentage is highly variable since the amount of membrane preserved after excision varies between patches. Thus, it is unclear if the missing 56% of the ciliary current originated from the disconnected cilia membrane or near the cilia-plasma membrane junction. As found for the polycystin-2 channels in the pIMCD cilia, increasing internal calcium stimulated multiple open polycystin-2 channels in the inside-out patch configuration of HEK-293 cilia (Figure 6E). Thus, ciliary polycystin-2 currents on *PKD2-GFP* overexpressing HEK-293 cells reproduces the ion selectivity and internal calcium sensitization as found in the native polycystin-2 channels of pIMCD cilia.

## Discussion

### *Arl13b-EGFP^tg^:cPkd1* and *Arl13b-EGFP^tg^:cPkd2* as new mouse models for cilia visualization during ADPKD progression

We confirmed cyst progression upon reduction of *Pkd1* or *Pkd2* in adult renal collecting duct epithelia. Because the mice also expressed the *Arl13b-EGFP^tg^* transgene, we could compare the effects of reduced polycystins on cilia formation. Conditional ablation of either gene did not block the formation of cilia in adult collecting ducts, consistent with findings in the embryonic node, where constitutive global repression of polycystin-2 had no effect on cilia number (Field et al., 2011). Unexpectedly, we observed elongated and twisted cilia, a feature that became more pronounced as the cystic phenotype progressed. These data support the hypothesis that ciliary polycystin-1 and polycystin-2 are essential to the maintenance of normal renal tubular cell differentiation. It is important to point out that the observed altered ciliary morphology and cyst formation are not necessarily causally related. Currently, we do not understand how loss of polycystins affect cilia morphology, but hypothesize that they alter ciliary transport or modification of proteins shuttling through or sequestered within cilia.

### Polycystin-2 is primarily a monovalent channel in the cilium

A commonly held hypothesis is that polycystin-1 and polycystin-2 form a calcium-permeant channel directly involved in aberrant cytoplasmic calcium signaling (Pei, 2001). Previous work measuring reconstituted polycystin-2 channels from heterologous and native sources report conflicting voltage sensitivity and ion selectivity (Pablo et al., 2017). Here, by directly measuring channels in primary cilia, we have shown that polycystin-2 is an essential subunit for the outwardly rectifying current. In primary cilia of native kidney tubular epithelial cells, polycystin-2 current is relatively selective for monovalent cations (P_x_/P_Na_ = 2.4 and 1, for K^+^ and Na^+^, respectively), with comparatively little calcium permeation (Ca^2+^ ~ NMDG).

The cilium, like the dendrite of neurons, is a quasi-distinct compartment, enforced by >100 MΩ resistance between the volumes, >100 fold differences in surface areas (~10 pF vs ~100 fF), distinct proteins with distinct ion binding characteristics, and restricted diffusion. Nonetheless, since there is no membrane separation between compartments, it is important to measure these compartments separately, as we previously reported (DeCaen et al., 2013). The pIMCD and HEK whole-cell currents capture a distinct set of ion channels from those measured in the whole-cilium configuration. As confirmed by excised whole-cilium recordings, currents from the whole-cell and whole-cilium can be reliably separated. Currents from these membranes can also be distinguished by changing external cations (compare Figure 7A–C, Figure 7—figure supplement 1A–B with Figure 4B). The pIMCD whole-cell cationic currents (Figure 7B) consist of an inwardly rectifying potassium current (Kir), a voltage-gated calcium current (Ca_v_), and an outwardly-rectifying sodium current. In contrast, the pIMCD whole-cilium current is a non-selective outwardly-rectifying current dependent on polycystin-2 expression (Figure 4B).

Kleene and Kleene identified polycystin-2 as a large conducting ion channel (outward γ = 96 pS) of mIMCD-3 cells (Kleene and Kleene, 2017). mIMCD-3 are immortalized epithelial cells derived from the terminal portion of the inner medullary collecting duct of SV40 transgenic mice and have similar morphology to the primary IMCD epithelial cells used in our study. We observed a similar outward conductance (γ = 90–117 pS) in the cilia of primary collecting duct epithelial cells directly harvested from adult mice. Like the results reported from the mIMCD-3 cell lines, unitary and whole-cilium currents from the pIMCD primary cells were reduced or absent when polycystin-2 was knocked down with siRNAs or conditionally knocked-out in the whole animal. Both studies note the sensitization of the current by µM [Ca^2+^]. Based on these similarities, it is likely that we are describing the same ciliary polycystin-2 channel in these cell types. Both studies agree that the polycystin-2 cilia conductance is most selective for potassium but differ in estimates of sodium and calcium permeability (P_x_/P_K_ = 1: 0.14: 0.55 for K^+^, Na^+^ and Ca^2+^, respectively) (Kleene and Kleene, 2017) compared to P_X_/P_K_ = 1: 0.4: 0.025 for K^+^, Na^+^ and Ca^2+^, respectively, in this study. To compare readily with our previous work describing other ciliary polycystin channels (Shen et al., 2016; DeCaen et al., 2016; DeCaen et al., 2013), we report P_X_/P_Na_ = 2.4, 1, and 0.06 for K^+^, Na^+^, and Ca^2+^, respectively.

The relative permeabilities of Na^+^ and K^+^ are relatively consistent between Kleene and Kleene, Pavel et al, and our results. The ~20 fold difference in P_Ca_/P_K_(0.55 vs 0.025) most probably arises from the linear extrapolation by Kleene et al to obtain the E_rev_. Other differences may be relevant: we recorded from primary tubule cells (pIMCD), not from immortalized cells (mIMCD-3; near-triploid karyotype) (Battini et al., 2008). Second, external [Ca^2+^] was chelated to <1 nM (and Mg^2+^<10 µM) in our solutions in which inward monovalent currents were measured. Kleene and Kleene added 2 mM Mg^2+^ and varying [Ca^2+^] in both internal and external conditions (Kleene and Kleene, 2017). Finally, the method utilized by Kleene and Kleene envelops the cilium and its base within the recording pipette, thus including some plasma membrane (Kleene and Kleene, 2012). Here, we patch only ciliary membrane, albeit with some of its base removed (see Video 1) (DeCaen et al., 2013). To help refine our measurement, we estimated the relative permeability based on the extrapolated E_rev_ from our single channel currents (Figure 5—figure supplement 2D). The ion selectivity results from our single channel and whole-cilium measurements are consistent, which strengthens our conclusions regarding the rank order of cation selectivity; K^+^>Na^+^>>Ca^2+^.

It is important to note that neither we nor the Kleene group found inward calcium-mediated single channel events from potentials ranging from 0 mV to −100 mV (personal communication with Steven Kleene, Univ. of Cincinnati). However, we were able to resolve unitary single channel events under non-physiological conditions in which the external calcium was high (110 mM) and the cilium’s membrane potential was very hyperpolarized (more negative than −120 mV). Thus, there would be little calcium influx into cilia under physiological conditions since the resting cilia membrane potential is only −18 mV (Delling et al., 2013). Nonetheless, aberrant calcium signaling has been observed from cells expressing mutant polycystin-2 channels and interpreted as due to polycystin-2 function as an ER calcium-release channel (Cai et al., 2004; Ćelić et al., 2012), a function we have not investigated. Since Ca^2+^ changes in the cytoplasm propagate into cilium (Delling et al., 2016), we cannot rule out the possibility that polycystin-2 in the ER alters [Ca^2+^]_cilium_. Also, although polycystin-2’s Ca^2+^ conductance is small, the cilium is a < 1 fL restricted space in which localized proteins might be influenced directly by occasional ciliary polycystin-2 channel Ca^2+^ flux. However, it should not be overlooked that the major consequence of polycystin-2’s selectivity is to depolarize the cilium and raise [Na^+^]_cilium_. If Na^+^/Ca^2+^ exchangers or Na^+^ -dependent kinases are found in cilia, polycystin-2 activity could underlie a slow, cumulative signal via calcium changes and/or kinase activity.

### Potential polycystin-2-specific function in kidney

Renal epithelial cilia are exposed to urine, which contains varying ion concentrations as a function of position in the nephron. Human and murine distal collecting duct epithelial cells are exposed to high external concentrations of potassium (90–260 mM) and sodium (53–176 mM) ions, which contributes to the hyperosmolarity of urine (390–650 mOsm being considered normal, but can vary beyond this range depending on hydration state) (Callís et al., 1999; Sands and Layton, 2014). Ciliary influx through polycystin-2, driven by these extreme extracellular concentrations of Na^+^ and K^+^ ions, may depolarize the plasma membrane sufficiently to activate voltage-gated calcium channels present in the plasma membrane. A recent computational study finds that opening of single ciliary polycystin 2-L1 channel (~150 pS) is sufficient to trigger action potentials in the soma of cerebrospinal fluid-contacting neurons at standard concentrations in blood plasma (Orts-Del'Immagine et al., 2016). Future electrophysiological studies using current clamp will determine whether the activation of ion channels in the ciliary membrane is sufficient to depolarize the plasma membrane of pIMCD cells, if there is any polycystin-1-dependent polycystin-2 function that has different ion selectivity or permeability compared to polycystin-1-independent function, or if there are more direct consequences of polycystin-2 expression for other ciliary compartment proteins.

### Ciliary polycystin-2 channels are activated by internal calcium – relevance to kidney

We have demonstrated that extraciliary Ca^2+^ can block monovalent conductance through polycystin-2, a common phenomenon in selective and non-selective cation channels, and responsible for the anomalous mole fraction effect (Eisenman et al., 1986; Friel and Tsien, 1989; Sauer et al., 2013). This effect is likely due to a relatively higher affinity for Ca^2+^ ions in the pore (IC_50_ = 17 mM), thus blocking the channel to the inward passage of Na^+^ and K^+^ ions. Recently, polycystin-2 structures were captured in the ‘single’ and ‘multi-ion mode' states; 20 mM Ca^2+^ and 150 mM Na^+^ were present during protein purification. Ca^2+^ bound at the entrance of the selectivity filter suggests either a low Ca^2+^ conducting state or a blocked state, in which Ca^2+^ ions prevent Na^+^ ion permeation in the multi-ion mode (Grieben et al., 2017; Wilkes et al., 2017). These studies provide a structural context for the anomalous mole fraction effects we observe in polycystin-2 currents from the pIMCD cilia and those reported by other groups measuring polycystin-2 channels from other preparations (Arif Pavel et al., 2016; Cai et al., 2004; Koulen et al., 2002; Vassilev et al., 2001). What, if any, effect might anomalous mole fraction effects have on the ciliary polycystin-2 channel? In contrast to the physiological, typically tightly-controlled interstitial [Ca^2+^] (~1.8 mM), urinary [Ca^2+^] in humans and mice is highly variable (5–20 mM) during normal diurnal activity (Foley and Boccuzzi, 2010). Urinary Ca^2+^ may have physiologically-relevant effects in dynamically limiting the polycystin-2 monovalent current through the cilium. When calcium-wasting occurs (~15 mM tubular [Ca^2+^]), more than half of Na^+^ and K^+^ influx through polycystin-2 would be antagonized (see Figure 5—figure supplement 1). Thus, ciliary polycystin-2 would be most active during low [Ca^2+^] in the tubule urine. On the other side of the cilia membrane, shifting the internal [Ca^2+^] from resting levels (600 nM) to 30 μM activates the polycystin-2 current by increasing the open probability and the total inward conductance ~10 fold. Internal Ca^2+^-dependent potentiation has been reported in polycystin-2 channels reconstituted from the ER into bilayers, but these channels inactivate at [free-Ca^2+^]_in _concentrations > 1 μM unless mutated at C-terminal phosphorylation sites (Cai et al., 2004). Previous measurements of resting [free-Ca^2+^]_in_ from RPE cilia (580 nM) (Delling et al., 2013) and from mouse embryonic node cilia (305 nM) (Delling et al., 2016) are 3–6 times higher than in the cell body. Primary cilia [free-Ca^2+^]_in_ increases to levels greater than 1 μM when mIMCD-3 cells (and other cell types) are exposed to flow or shear stress (Delling et al., 2013; Su et al., 2013). Recent work has demonstrated that primary cilia are not Ca^2+^-responsive mechanosensors themselves; rather, mechanically-induced calcium waves are initiated from other locations to raise ciliary [Ca^2+^] (Delling et al., 2016). Thus, increasing cytoplasmic [free-Ca^2+^] by mechanical or other stimuli, may increase cilioplasmic calcium and potentiate ciliary polycystin-2 channel activity. Consistent with the cilia channel recordings made by Kleene and Kleene (Kleene and Kleene, 2017), a 50x increase in the cilioplasmic calcium reduces the voltage threshold required to activate polycystin-2 from our pIMCD cilia. If the cilia membrane potential of collecting duct cells is as depolarized as the cilia of RPE cells (−18 mV), then we can expect an ~10 x increasing polycystin-2 opening when cytoplasmic calcium waves reach the cilioplasm.

### Polycystin-2 and polycystin 2-L1, independently form ion channels in primary cilia in disparate tissues

Previously we characterized the ciliary current from retina pigmented epithelial cells (RPE) and mouse embryonic fibroblasts (MEF) and demonstrated that they require the polycystin family proteins, polycystin 1-L1 and polycystin 2-L1, based on attenuation of the cilia current by siRNA and genetic ablation of these two genes (DeCaen et al., 2013). Using similar methodology, including cilia electrophysiology from primary collecting duct cells, we have determined that polycystin-2 is at least a component of I_cilia_ from these cells. Thus, polycystin-2 and polycystin 2-L1 may be ciliary ion channels inhabiting distinct cellular tissues. Although the single channel conductance and sensitization of the RPE and MEF *Pkd1-L1/2-L1*-encoded cilia channel (Inward γ_Na_ = 80 ± 3 pS) (DeCaen et al., 2013) is similar to the pIMCD cilia polycystin-2 channel (Inward γ_Na_ = 89 ± 4 pS), their Ca^2+^ selectivity is distinct. Also, we showed here that the ciliary pIMCD polycystin-2 channel is blocked by external Ca^2+^ but is sensitized by high (EC_50_ = 1.2 μM) internal Ca^2+^,~10 times the typical resting cytoplasmic concentration. Heterologous polycystin 2-L1 channels are also sensitized by increases in [Ca^2+^]_in_ (although the sensitivity range has not been determined) based on cytoplasmic Ca^2+^ uncaging studies and expected Ca^2+^ accumulation in whole cell experiments (DeCaen et al., 2016). However, a key difference between pIMCD (polycystin-2) and RPE (polycystin 2-L1) ciliary channels are that polycystin 2-L1 channels preferentially conduct Ca^2+^ (P_Ca_/_Na_ = 6–19) (DeCaen et al., 2013; DeCaen et al., 2016) over monovalent ions. Mutagenesis studies of heterologous polycystin 2-L1 channels has demonstrated that Ca^2+^ permeation is at least partly due to an additional glutamate residue (D525) on the external side of the selectivity filter, not present in polycystin-2 (DeCaen et al., 2016). The physiological implications for the differential cilia expression of polycystins and attendant differences in ion selectivity is not known.

### Polycystin-2 structural considerations

Soon after these studies, polycystin-2 core structures were solved using single-particle electron cryo-microscopy in which the polycystin-2 channels formed a homotetrameric structure (Grieben et al., 2017; Shen et al., 2016), independent of coiled-coil domains (originally posited to form an interaction motif with polycystin-1 subunits [Qian et al., 1997]). As reported (Shen et al., 2016), replacement of the polycystin 2-L1 filter with that of polycystin-2 conferred monovalent selectivity to the otherwise Ca^2+^-permeant polycystin 2-L1 channel. However, it is important to note that the native ciliary polycystin-2 channel’s ion selectivity, as described here, is not completely recapitulated in the polycystin-2 filter chimera, where single channel Ca^2+^ conductance was~27 fold smaller than K^+^ (γ_Ca_ = 8 ± 2 pS, twice as large as the native cilia polycystin-2 channels, γ_Ca_ = 4 ± 1 pS). Nonetheless, the native polycystin-2 cilia channel and the polycystin-2 filter chimera share similar ion selectivity, where K^+^ is favored over Na^+^ and Ca^2+^ as reflected in the magnitudes of single channel conductance (pIMCD cilia γ_K_ = 144 ± 6 pS, γ_Na_ = 89 ± 4 pS; polycystin-2 chimera γ_K_ = 218 ± 3 pS, γ_Na_ = 139 ± 3 pS) and relative permeability (P_x_/P_Na_ pIMCD cilia = 2.4: 1: 0.06 and polycystin-2 chimera = 2.2: 1: 0.5 for K^+^, Na^+^ and Ca^2+^ respectively).

### Loss of polycystin-1 does not alter polycystin-2 ciliary trafficking or polycystin-2 mediated ciliary currents

Based on the two-hit hypothesis of ADPKD, inherited haploinsufficiency of either polycystin-1 and polycystin-2 and a second acquired somatic mutation is required for disease progression. It was reported that interaction between polycystin-1 and polycystin-2 is required for cell plasma membrane trafficking of the complex (Chapin et al., 2010; Gainullin et al., 2015; Hanaoka et al., 2000), but their interdependence for ciliary localization is controversial. In some studies, polycystin-2 is absent from primary cilia without polycystin-1 or *vice versa *(Gainullin et al., 2015; Kim et al., 2014), while others showed polycystin-2 traffic to cilia is independent of polycystin-1 (Cai et al., 2014; Geng et al., 2006; Hoffmeister et al., 2011). Our data support the view that polycystin-2 can traffic to primary cilia of pIMCD cells in the absence of polycystin-1. The differences with some of the previous reports may due to the different experimental systems. We employed isolated pIMCD cells with few or no passages, while others used cell lines (mIMCD3, HEK-293, LLC-PK1, or Renal Cortical Tubule Epithelial; RCTE) or cells overexpressing proteins. Another difference is that our mice express the cilia marker *Arl13b-EGFP*, which might affect ciliary trafficking. There are no other studies reporting this mouse model to date.

Based on biochemical data and initial whole-cell electrophysiology, coexpression of polycystin-1 and polycystin-2 were reported to be necessary and sufficient to form heteromeric Ca^2+^-permeant channels in the cell soma plasma membrane (Hanaoka et al., 2000). However, we have noted a dearth of recordings in the literature, and of the few published, there are many inconsistencies. Based on the data here, and the selectivity measured from the polycystin-2 gating mutant (F604P) (Arif Pavel et al., 2016), polycystin-2 is primarily a monovalent channel with selectivity K^+^ > Na^+^, whose current is blocked by extracellular Ca^2+^. It resembles many TRP channels in being slightly outwardly rectifying under physiological conditions, with a large single channel conductance (~100 pS). Polycystin-1 and polycystin-2 were believed to form a channel by association with their C-terminal coiled-coil domain (Qian et al., 1997). In contrast, recent structures of polycystin-2 homotetramers channels suggest that it can form a pore in the absence of the polycystin-2-coiled-coil domain and without polycystin-1 subunits (Grieben et al., 2017; Shen et al., 2016; Wilkes et al., 2017). Furthermore, removal of the conserved coiled-coil does not alter homomeric functional assembly of the related polycystin 2-L1 channel (DeCaen et al., 2016; Li et al., 2002).

Our genomic PCR data (Figure 1—figure supplement 2A and E) cannot rule out the possibility that not all pIMCD cells completely lacked polycystin-1, although the more functionally-relevant patch clamp results show no current differences (Figure 4) between doxycycline-treated and untreated groups. Our conditional knockout mice are inducible knockouts of *Pkd1* or *Pkd2* in renal tubular epithelial cells in the Pax8rtTA; TetO-cre system, but not all tubule cells are knock-out cells (e.g., the S3 straight segment, where Cre activity was largely absent [Ma et al., 2013]). Non-tubule cells like fibroblasts, interstitial cells, and endothelial cells may also affect the purity of the cells we isolated. Our conclusions are primarily based on patch clamp electrophysiology, which is more sensitive and functionally relevant than western blots, tagging, or immunohistochemistry. We ablated polycystin-1 and still observe the same magnitude of polycystin-2 currents from the discrete primary cilium (Figure 4B). If polycystin-1 is necessary – either as a polycystin-2 chaperone or part of the functional channel complex – its impact on polycystin-2 ion channels should have been detected with electrophysiological recordings. It is important to note that our doxycycline-dependent ablation of polycystin-1 (or polycystin-2) is sufficient to produce the cystic kidney phenotype in these mice. Thus, reduced levels, whether complete or not, are sufficient for the observed pathophysiological changes.

As a final caveat, our results cannot exclude the possibility that polycystin-1 may associate with polycystin-2 in the cilium or perhaps in the membrane of the endoplasmic reticulum or Golgi. It is also possible that polycystin-1 may still modulate ciliary currents, perhaps indirectly as a receptor for extracellular ligands, or through direct association. However, our findings demonstrate that polycystin-1 is not essential for basal activity of polycystin-2 in primary cilia of pIMCD cells. Further work will likely refine the mechanism of the two-hit model of ADPKD progression.

### Polycystin-2 is not a constitutively active ion channel in the plasma membrane

In previous work, we established that polycystin 2-L1 can form a constitutively active ion channel in the plasma membrane and in primary cilia (DeCaen et al., 2013, DeCaen et al., 2016). We also reported that polycystin-2 did not appear to function on the plasma membrane, where HEK-293 and CHO cells transiently overexpressing polycystin-2, with or without polycystin-1, have the same level of plasma membrane cation currents observed in untransfected cells (DeCaen et al., 2013; Shen et al., 2016). In this manuscript, we present several lines of evidence suggesting that polycystin-2, unlike polycystin 2-L1, does not constitutively function in the plasma membrane in kidney epithelial cells. First, conditional knockout of polycystin-2 does not alter the major cation currents found in the plasma membrane of primary collecting duct epithelial cells. Second, we did not observe differences in plasma membrane current measured from HEK-293 cells stably-expressing *PKD2-GFP* in parental cells. While it is possible that polycystin-2 in the plasma membrane could be stimulated by a ligand such as Wnt3a, Wnt9b, and triptolide, we have not been able to reproduce activation of heterologous polycystin-2 with these reagents (Kim et al., 2016; Leuenroth et al., 2007; Ma et al., 2005). Functional polycystin-2, heterologously expressed in the plasma membrane of *Xenopus laevis* oocytes, required an F604P mutation near its intracellular gate (Arif Pavel et al., 2016), similar to mutations required for TRPML plasma membrane function (Grimm et al., 2012; Xu et al., 2007b). The polycystin-2 F604P current was selective for potassium and sodium, but blocked by extracellular calcium and magnesium. Our attempts to record overexpressed polycystin-2 F604P membrane currents in mammalian cells have been unsuccessful (Shen et al., 2016), but such expression in *Xenopus* oocytes has been reproduced (unpublished data, courtesy of Michael Sanguinetti, Univ. Utah). What is clear is that *wt* polycystin-2 has no measurable constitutive activity in the plasma membrane in either mammalian or *Xenopus* expression systems.

### Polycystin-2-GFP cilia recording for detection of heterologous expression

Other groups have observed epitope-labeled polycystin-1 and polycystin-2 in HEK-293 cell primary cilia (Gerdes et al., 2007; Lauth et al., 2007). C-terminal GFP-tagged polycystin-2 enriches in primary cilium when stably expressed in HEK-293 cells. We observed that N-terminal GFP-tagged polycystin-2 fails to localize to the cilium, suggesting that the sensor in this position interferes with trafficking. It is possible that the N-terminal GFP tag may interfere with the amino-terminal cilia-localization sequence (R_6_VXP)(Geng et al., 2006) and likewise, the C-terminal GFP tag may interfere with the ER retention sequence found in the C-terminus (Cai et al., 1999). However, since we have demonstrated that native (untagged) polycystin-2 is functionally expressed in cilia of collecting duct epithelia, the C-terminally-tagged polycystin-2 over-expressed in HEK-293 cilia appears to properly localize. Like many overexpressed ion channels, we observed a high amount of GFP fluorescence from N- and C-terminally-tagged polycystin 2-L1 within intracellular compartments. Polycystin-2 in the ER has been shown to be sensitized by cytoplasmic calcium, triggering Ca^2+^-induced Ca^2+^ release, possibly through direct interaction with the IP3R channel (Koulen et al., 2002; Vassilev et al., 2001). However, we did not examine polycystin-2 function in the ER or how cilia polycystin-2 may alter intracellular store calcium release. Future work should determine if polycystin-2 channels function in ER membranes of native tissue and if differential localization confers unique channel properties. These findings present new avenues to study mutant forms of polycystin-2 that cause ADPKD. This method could be used to determine which ADPKD forms of polycystin-2 are gain-of-function or loss-of-function, and perhaps alter channel trafficking to cilia. Ultimately, outcomes from these studies could form a rational basis for polycystin-2 dysregulation in ADPKD and enhance our basic understanding of ciliary ion channel function in cell biology.

## Materials and methods

### Electrophysiology

All electrophysiology reagents used were manufactured by either Sigma Aldrich (St. Louis, MO) or Life Technologies (Carlsbad, CA). . Ciliary ion currents were recorded using borosilicate glass electrodes polished to resistances of 14–18 MΩ using the cilium patch method previously described (DeCaen et al., 2013). Holding potentials were −60 mV unless otherwise stated. The pipette standard internal solution (SIS) contained (in mM): 90 NaMES, 10 NaCl, 10 HEPES, 10 Na_4_-BAPTA (Glycine, N, N'-[1,2-ethanediylbis(oxy-2,1-phenylene)]bis[N-(carboxymethyl)]-,tetrasodium); pH was adjusted to 7.3 using NaOH. Free [Ca^2+^] was estimated using Maxchelator (Bers et al., 1994) and adjusted to 100 nM by adding CaCl_2_. Standard bath solution (SBS) contained 140 NaCl, 10 HEPES, 1.8 CaCl_2_; pH 7.4. Unless otherwise stated, ‘whole cilia’ ionic currents were recorded in symmetrical [Na^+^]. All solutions were osmotically balanced to 295 (±6) mOsm with mannitol. Data were collected using an Axopatch 200B patch clamp amplifier, Digidata 1440A, and pClamp 10 software. Whole-cilium and excised patch currents were digitized at 25 kHz and low-pass filtered at 10 kHz. To accurately measure membrane reversal potential, five current pulses from voltage ramps were averaged. Extra-membrane conditions were changed using a Warner Perfusion Fast-Step (SF-77B) system in which the patched cilia and electrode were held in the perfusate stream. Data were analyzed by Igor Pro 7.00 (Wavemetrics, Lake Oswego, OR). The reversal potential, E_rev_ was used to determine the relative permeability of K^+^, Na^+^ and NMDG (P_X_/P_Na_) using the following equation:

$$\frac{P_{X}}{P_{Na}}=\frac{\alpha_{Nae}}{\alpha_{Xe}}\left[exp(\frac{\Delta E_{rev}}{RT/F})\right]$$

where E_rev_, α, R, T and F are the reversal potential, effective activity coefficients for the cations (i, internal and e, external), the universal gas constant, absolute temperature, and the Faraday constant, respectively. The effective activities (α_x_) were calculated using the following equations:

$$\alpha_{x}=\gamma_{x}[X]$$

where γ_x_ is the activity coefficient and [X] is the concentration of the ion. For calculations of membrane permeability, activity coefficients (γ) were calculated using the Debye-Hückel equation: 0.79, 0.72, 0.30 and 0.24 correspond to Na^+^, K^+^, Ca^2+^ and NMDG^+^, respectively (γ for NMDG^+^ from (Ng and Barry, 1995). To determine the relative permeability of Ca^2+^ to Na^+^, the following equation was used:

$$\frac{P_{Ca}}{P_{Na}}=\frac{\left\{\alpha_{Nai}[exp(\frac{E_{rev}F}{RT})][exp(\frac{E_{rev}F}{RT})+1]\right\}}{4\alpha_{Cae}}$$

*E*_rev_ for each condition was corrected to the measured liquid junction potentials (−4.4 to 3.4 mV).

For the experiments shown in Figure 7 and Figure 5—figure supplement 1A, the internal pipette saline contained 90 mM NaCl, 10 HEPES and 5 Na_4_-BAPTA and pH was adjusted with NaOH. The extracellular bath solution contained 110 mM X-Cl, 10 HEPES and pH was adjusted with X-OH, where X corresponds to the cation tested (Na^+^, K^+^, Ca^2+^, NMDG^+^). Monovalent-based extracellular solutions contained 0.1 mM EGTA to remove residual divalent cations. The NaMES-based extracellular solution contained: 110 NaMES (sodium methanesulfonate); 10 HEPES; 0.1 EGTA and pH was adjusted with Tris base (tris(hydroxymethyl)aminomethane). For ‘on-cilia’ single channel recording, the resting membrane potential was neutralized with a high K^+^ bath solution that contained: 110 KCl, 20 NaCl, 10 HEPES, 1.8 CaCl_2_, and adjusted to pH 7.4 using KOH. To test the inward single channel conductance, the intracellular pipette solution was replaced with one of the above extracellular solutions. For ‘inside-out’ cilia recordings, the pipette (extra-ciliary) solution contained SBS and the bath (intraciliary) solution contained 150 NaCl, 10 HEPES, 5 EGTA and free [Ca^2+^] adjusted by adding CaCl_2_. When a cilium was excised from the cell, the severed end of the cilium commonly re-seals itself, which isolates the intraciliary membrane from the bath and limits the effect of bath applied exchange on the inside of the cilium. To avoid this, excised cilia patches were briefly pressed against the surface of a bead made of Sylgard 184 (Dow Corning) to rupture the cilia at the opposing end. In Figure 6—figure supplement 1D, the conductance measurement was made by dividing the concatenated single channel currents against the holding potential. In Figure 5—figure supplement 1B, we correct for rundown of the cilia current (I_corrected_) by fitting the control (nominally calcium-free period) inward current to a linear equation (y = mx + b). The difference between I_Ca_ (measured with calcium present) and I_corrected_ was then normalized: [(I_Ca_ − I_corrected_)/I_corrected_]×100. The potency of inward sodium current block was determined by fitting the percent inward current block and calcium concentration relationship to the Hill equation:

$$y=I_{minimum}+\frac{\left(I_{maximum}-I_{minimum}\right)}{1+{\left[\frac{IC_{50}}{Ca_{e}}\right]}^{K}}$$

Where I_minimum_ and I_maximum_ are the minimum and maximum response, IC_50_ is the half-maximum inhibition, and K is the slope factor.

### Antibodies, reagents and mice

Mouse monoclonal antibody against GAPDH was from Proteintech. Rabbit polyclonal antibodies against acetylated tubulin (Lys40) was purchased from Cell Signaling Technology (Danvers, MA). Chicken polyclonal antibody against GFP was from Aves Labs (Tigard, OR). Doxycycline and Fluoshield with 1,4-Diazabicyclo [2.2.2] octane were from Sigma-Aldrich; Hoechst 33342 was from Life Technologies. Rabbit polyclonal anti-mouse polycystin 2 (OSP00017W) and rabbit polyclonal anti-mAQP2 (PA5-3800) were from Thermo Fisher (Waltham, MA).

The *Pax8^rtTA^; TetO-cre; Pkd1^fl/fl^; Pax8^rtTA^; TetO-cre; Pkd2^fl/fl^* and *Arl13b-EGFP^tg^* mice have been previously described(Ma et al., 2013)^,41^. *Pax8^rtTA^; TetO-cre* and *Pkd1^fl/fl^; Pax8^rtTA^; TetO-cre; Pkd2^fl/fl^* mice were obtained from the Somlo lab (Yale University). *Arl13b-EGFP^tg^:cPkd1 or Arl13b-EGFP^tg^:cPkd2* mice were generated by breeding *Pax8^rtTA^; TetO-cre; Pkd1^fl/fl^ or Pax8^rtTA^; TetO-cre; Pkd2^fl/fl^* with *Arl13b-EGFP^tg^* mice. The genotype was determined by PCR with the following primers. *Pax8^rtTA^* with PCR product ~600 bp: IMR7385-CCATGTCTAGACTGGACAAGA; IMR7386 –CTCCAGGCCACATATGATTAG. *TetO-Cre* with PCR product ~800 bp: TetO-CMV-5'- GCAGAGCTCGTTTAGTGAAC; Cre-R-TCGACCAGTTTAGTTACCC. *Pkd1^fl/fl^* with PCR product ~500 bp (wild type ~300 bp): ND1 Lox 5'-CACAACCACTTCCTGCTTGGTG; ND1 Lox 3'-CCAGCATTCTCGACCCACAAG. *Pkd2^fl/fl^* with PCR product ~450 bp (wild type ~300 bp): D2loxF1- GGGTGCTGAAGAGATGGTTC; D2loxR1- TCCACAAAAGCTGCAATGAA. *Arl13b-EGFP^tg^* with PCR product ~700 bp (wild type ~400 bp): 83940- TGCAACTCTATATTCAGACTACAG; 84608-GTGGACATAATGGTCCCATTAAGC; Transgene 2562-CATAGAAAAGCCTTGACTTGAGGTTAG. Mice were bred and housed in pathogen-free conditions with access to food and water in the Animal Care Facility. All experimental procedures were approved by the Boston Children’s Hospital Animal Care and Use Committee (IUACUC).

### Cell culture

Primary epithelial cells were cultured from dissected kidney collecting ducts of transgenic mice(Delling et al., 2016). Inner medullae were removed from the kidney and disassociated using a Dulbecco’s phosphate buffered solution (DPBS) containing 2 mg/ml collagenase A and 1 mg/ml hyaluronidase. After mechanical disassociation on ice, medullary cells were cultured in Dulbecco’s modified essential medium (DMEM) supplemented with 10% fetal bovine serum (FBS) and 100 units/ml penicillin/100 μg/ml streptomycin. Cilia were patched from cells within 6 days after isolation and within one passage. siRNA knockdown efficiency was monitored for each experiment with a ‘scrambled’ silencer negative control 1 siRNA (Life Technologies). For generation of *PKD2-GFP* and *GFP-PKD2* stable cell lines, C-terminal or N-terminal GFP-tagged polycystin-2 was generated by subcloning *PKD2* cDNA into a modified pWPXLd vector. Puromycin (2 μg/ml) was used to screen stable cell lines, and these cells were tested for mycoplasma contamination. The *PKD2* (NM_000297) human cDNA ORF Clone was purchased from Origene (Rockville, MD).

### Genomic PCR for pIMCD cells isolated from *Arl13b-EGFP^tg^:cPkd1* mice

Genomic DNA was extracted from the pIMCD cells isolated from *Arl13b-EGFP^tg^:cPkd1* mice (with and without doxycycline treatment). To genotype the *Pkd1* alleles, primer 1 (P1), 5′-CCGCTGTGTCTCAGTGCCTG −3′, and P2, 5′-CAAGAGGGCTTTTCTTGCTG −3′, were used to detect the floxed allele (~400 bp) and the *wt* allele (~200 bp); while P1, 5′-CCGCTGTGTCTCAGTGCCTG −3′, and P3, 5- ATTGATTCTGCTCCGAGAGG −3′, were used to detect the *Deletion* alleles (Deletion of exons 2–4 of *Pkd1*) of *Del* (~650 bp) and the non-Del (~1.65 kb).

### Immunocytochemistry, confocal microscopy and structured illumination microscopy (SIM)

Cells were fixed with 4% PFA, permeabilized with 0.2% Triton X-100, and blocked by 10% bovine serum albumin in PBS. Cells were labeled with the indicated antibody and secondary fluorescently-labeled IgG (Life Technologies) and Hoechst 33342. Confocal images were obtained using an inverted Olympus FV1000 with a 60x silicon oil immersion, 1.3 N.A. objective. Super resolution images using the SIM method where captured under 100x magnification using the Nikon Structured Illumination Super-Resolution Microscope (N-SIM) with piezo stepping. Confocal and SIM images were further processed with FIJI ImageJ (NIH).

### Immunohistochemistry and cyst parameters

*Arl13b-EGFP^tg^:Pax8^rtTA^;TetO-cre;Pkd1^fl/fl^* and *Arl13b-EGFP^tg^:Pax8^rtTA^;TetO-cre;Pkd2^fl/fl^* mice were induced with 2 mg/ml doxycycline in drinking water supplemented with 3% sucrose for 2 weeks from P28 to P42. Mice were anesthetized and perfused with 4% (wt/vol) paraformaldehyde at 8 weeks and 16 weeks after removal of doxycycline water. Kidneys were harvested and fixed with 4% paraformaldehyde at 4°C overnight, and embedded in paraffin. Sagittal kidney sections were stained with hematoxylin and eosin (H and E) and examined by light microscopy. All kidneys were photographed under the same magnification. ImageJ analysis software was used to calculate the cyst index (equal to the cumulative area of cysts within the total area of the kidney). For immunofluorescence of acetylated tubulin, a Leica VT1000S vibrating blade microtome was used for sectioning, kidney sections permeabilized with 0.5% TX100/PBS pH 7.4 overnight, and blocked with BlockAidTM solution for 5–8 hr. Sections were washed X3 in PBS, primary antibodies diluted in blocking solution, and sections incubated overnight at 4°C. After slides were washed X3 with PBS, goat anti-chicken/anti-rabbit fluorescent-labeled secondary antibodies were applied at room temperature overnight. Hoechst 33342 nuclear dye was incubated with sections for 1 hr. Sections were washed X3 with PBS, mounted in Fluoshield_TM_ with 1,4-Diazabicyclo [2.2.2] octane and imaged with an inverted Olympus FV1000; silicon oil immersion 60x, 1.3 N.A. objective. Images were further processed and cilia length was measured using Fiji ImageJ (NIH).

### Inhibition and detection of transcripts

Approximately 200,000 primary cells were transfected with 100 pM of siRNAs (ThermoFisher) and 10 μl Lipofectamine RNAiMAX (Life Technologies) in a 9.5 cm^2^ dish. A list of the siRNAs is described in Table 1. At least 48 hr after transfection, half the cells were placed onto glass coverslips for electrophysiology, while the other half were lysed in TRIzol reagent (Ambion) for RNA extraction according to the manufacturer’s instructions. RNA was reverse transcribed using the SuperScript reverse transcription kit (ThermoFisher Scientific). Gene-specific primers were designed using Primerbank (http://pga.mgh.harvard.edu/primerbank/)(Spandidos et al., 2008). Transcripts were amplified by PCR and expression was visualized by agarose gel electrophoresis. Sequences for gene-specific primers are listed in Table 3.

**Table 3.** Primers used to detect gene expression using qPCR*.

Gene, m, *Mus musculus; H, human*	Forward primer 5’−3’	Reverse primer 5’−3’
m*Pkd1*	CTGGGTGATATTTTGGGACGTAA	GCGTGGCAGTAGTTATCTGCT
m*Pkd1-L1*	ATGCCACTCTTGAAGTGAGCA	CCAGGCAGTGTATCTTCTTCCA
m*Pkd2*	TACAGCGACCCTCCTTCCC	CCTCTGATGCTCCGACAGATATG
m*Pkd2-L1*	CGTGGACATACCATTCCCAGA	ACGGAGAAGTCGATAAAGACCA
m*Trpv4*	ATGGCAGATCCTGGTGATGG	GGAACTTCATACGCAGGTTTGG
hPKD2	CGTGCCCCAGCCCAGTC	TTCCAGTACAGCCCATCCAATAAG

^*^Athanasia Spandidos, Xiaowei Wang, Huajun Wang, Stefan Dragnev, Tara Thurber and Brian Seed: A comprehensive collection of experimentally validated primers for Polymerase Chain Reaction quantitation of murine transcript abundance. B*MC Genomics* 2008, 9:633.

### Statistical analysis

Statistical comparisons were made using two-tailed Student's *t*-tests using OriginPro software (OriginLab, Northampton MA). Experimental values are reported as the mean ± S.E.M. unless otherwise stated. Differences in mean values were considered significant at p<0.05.

## Additional files

Transparent reporting form
