# Supplementary material for: Polycystin-2 is an essential ion channel subunit in the primary cilium of the renal collecting duct epithelium
Source: eLife. 2018 Feb 14;7:e33183. doi: 10.7554/eLife.33183 (PMC5812715; doi:10.7554/eLife.33183)
Supplement: Transparent reporting form [file elife-33183-transrepform.docx]

***eLife’s* transparent reporting form**

We encourage authors to provide detailed information *within their submission* to facilitate the interpretation and replication of experiments. Authors can upload supporting documentation to indicate the use of appropriate reporting guidelines for health-related research (see [EQUATOR Network](http://www.equator-network.org/%20)), life science research (see the [BioSharing Information Resource](https://biosharing.org/)), or the [ARRIVE guidelines](http://www.plosbiology.org/article/info:doi/10.1371/journal.pbio.1000412) for reporting work involving animal research. Where applicable, authors should refer to any relevant reporting standards documents in this form.

If you have any questions, please consult our Journal Policies and/or contact us: [editorial@elifesciences.org](mailto:editorial@elifesciences.org).

**Sample-size estimation**

- You should state whether an appropriate sample size was computed when the study was being designed
- You should state the statistical method of sample size computation and any required assumptions
- If no explicit power analysis was used, you should describe how you decided what sample (replicate) size (number) to use

Please outline where this information can be found within the submission (e.g., sections or figure legends), or explain why this information doesn’t apply to your submission:

We did not pre-estimate effect sizes because of the exploratory nature of the experiments. In general, for quantification of cyst formation, at least 5 mice were used as shown in the figures for each group; for ciliary length quantification, no less than 16 cilia were used to analyze the data as noted in the figures for each group; for representative images or figures, the number of samples is noted in the figure legends; for patch clamp recordings, more than 4 primary cilia were recorded as noted in the figures.

**Replicates**

- You should report how often each experiment was performed
- You should include a definition of biological versus technical replication
- The data obtained should be provided and sufficient information should be provided to indicate the number of independent biological and/or technical replicates
- If you encountered any outliers, you should describe how these were handled
- Criteria for exclusion/inclusion of data should be clearly stated
- High-throughput sequence data should be uploaded before submission, with a private link for reviewers provided (these are available from both GEO and ArrayExpress)

Please outline where this information can be found within the submission (e.g., sections or figure legends), or explain why this information doesn’t apply to your submission:

For the western blots, confocal imaging, and example patch clamp recordings, the number of independent biological replicates is referenced in the figure legends. For the quantification of cyst formation, ciliary length, and patch clamp recordings, the number of samples is shown in the figures.

**Statistical reporting**

- Statistical analysis methods should be described and justified
- Raw data should be presented in figures whenever informative to do so (typically when N per group is less than 10)
- For each experiment, you should identify the statistical tests used, exact values of N, definitions of center, methods of multiple test correction, and dispersion and precision measures (e.g., mean, median, SD, SEM, confidence intervals; and, for the major substantive results, a measure of effect size (e.g., Pearson's r, Cohen's d)
- Report exact p-values wherever possible alongside the summary statistics and 95% confidence intervals. These should be reported for all key questions and not only when the p-value is less than 0.05.

Two-tailed Student's *t*-tests are used in the manuscript. Experimental values are reported as the mean ± s.e.m. unless otherwise stated in the figure legends. Differences in mean values were considered significant at P < 0.05.

Number (n) and P value are included in the figures.

Please outline where this information can be found within the submission (e.g., sections or figure legends), or explain why this information doesn’t apply to your submission:

(For large datasets, or papers with a very large number of statistical tests, you may upload a single table file with tests, Ns, etc., with reference to sections in the manuscript.)

**Group allocation**

- Indicate how samples were allocated into experimental groups (in the case of clinical studies, please specify allocation to treatment method); if randomization was used, please also state if restricted randomization was applied
- Indicate if masking was used during group allocation, data collection and/or data analysis

Please outline where this information can be found within the submission (e.g., sections or figure legends), or explain why this information doesn’t apply to your submission:

Groups were allocated by genotype, and each genotype was allocated by doxycycline treatment, or lack thereof, as indicated in figures, figure legends, and text.

**Additional data files (“source data”)**

- We encourage you to upload relevant additional data files, such as numerical data that are represented as a graph in a figure, or as a summary table
- Where provided, these should be in the most useful format, and they can be uploaded as “Source data” files linked to a main figure or table
- Include model definition files including the full list of parameters used
- Include code used for data analysis (e.g., R, MatLab)
- Avoid stating that data files are “available upon request”

Please indicate the figures or tables for which source data files have been provided:

Figure 4-Source Data 1-ciliary currents for siRNA screen of TRP proteins in cilia

Figure 4-Source Data 2-whole ciliary currents in polycystin-1 or polycystin-2 knockout primary cells

Figure 7-figure supplement 3-Source Data 1-ciliary currents of cells treated with PKD2-mRNA-targeted siRNAs
